# Development and psychometric properties of surveys to assess patient and family caregiver experience with care transitions

**DOI:** 10.1186/s12913-021-06766-w

**Published:** 2021-08-09

**Authors:** Joann Sorra, Katarzyna Zebrak, Deborah Carpenter, Theresa Famolaro, John Rauch, Jing Li, Terry Davis, Huong Q. Nguyen, Megan McIntosh, Suzanne Mitchell, Karen B. Hirschman, Carol Levine, Jessica Miller Clouser, Jane Brock, Mark V. Williams

**Affiliations:** 1 Westat, Rockville, Maryland USA; 2 Center for Health Services Research, University of Kentucky, Lexington, Kentucky USA; 3 Louisiana State University Health Shreveport, Shreveport, Louisiana USA; 4 Kaiser Permanente Southern California, Pasadena, California USA; 5 Boston Medical Center/Boston University School of Medicine, Boston, Massachusetts USA; 6 NewCourtland Center for Transitions and Health, University of Pennsylvania School of Nursing, Philadelphia, Pennsylvania USA; 7 United Hospital Fund, New York, New York USA; 8 Telligen, Greenwood Village, Colorado USA

**Keywords:** Transitional care, Patients, Patient experience, Caregivers, Surveys and questionnaires, Psychometrics, Quality of healthcare, Hospitals

## Abstract

**Background:**

The purpose of this study was to develop and administer surveys that assess patient and family caregiver experiences with care transitions and examine the psychometric properties of the surveys. The surveys were designed to ask about 1) the transitional care services that matter most to patients and their caregivers and 2) care outcomes, including the overall quality of transitional care they received, patient self-reported health, and caregiver effort/stress.

**Methods:**

Survey items were developed based on a review of the literature, existing surveys, focus groups, site visits, stakeholder and expert input, and patient and caregiver cognitive interviews. We administered mail surveys with telephone follow up to patients recently discharged from 43 U.S. hospitals. Patients identified the caregivers who helped them during their hospital stay (Time 1 caregiver) and when they were home (Time 2 caregiver). Time 1 and Time 2 caregivers were surveyed by telephone only. The psychometric properties of the survey items and outcome composite measures were examined for each of the three surveys. Items that performed poorly across multiple analyses, including those with low variability and/or a high missing data, were dropped except when they were conceptually important.

**Results:**

The analysis datasets included responses from 9282 patients, 1245 Time 1 caregivers and 1749 Time 2 caregivers. The construct validity of the three proposed outcome composite measures—Overall Quality of Transitional Care (patient and caregiver surveys), Patient Overall Health (patient survey) and Caregiver Effort/Stress (caregiver surveys) —was supported by acceptable exploratory factor analysis results and acceptable internal consistency reliability. Site-level reliability was acceptable for the two patient outcome composite measures, but was low for Caregiver Effort/Stress (< 0.70). In all surveys, the Overall Quality of Transitional Care outcome composite measure was significantly correlated with other outcome composite measures and most of the single-item measures.

**Conclusions:**

Overall, the final patient and caregiver surveys are psychometrically sound and can be used by health systems, hospitals, and researchers to assess the quality of care transitions and related outcomes. Results from these surveys can be used to improve care transitions, focusing on what matters most to patients and their family caregivers.

**Supplementary Information:**

The online version contains supplementary material available at 10.1186/s12913-021-06766-w.

## Background

Patient transitions in care from the hospital to post-acute settings or home continue to be fraught with potential gaps in care and services that can put patients at risk and overburden family caregivers [1, 2]. It is therefore important to identify the critical transitional care services or groups of services that matter most to patients and family caregivers and that yield the best outcomes. In a literature review on patient experience with healthcare, Wolf et al. [3] found that studies about patient experience focus on individualized care and tailoring of services to meet patients’ needs and engage them as partners in their care, which is integral to the principles and practice of patient- and family- centered care. Other research has found that better patient care experiences are associated with better clinical outcomes [4, 5], better patient safety culture within hospitals [6], and lower 30-day hospital readmission rates for acute myocardial infarction, heart failure, and pneumonia [7].

While patients’ experiences are increasingly recognized as a critical component of the overall quality of care, much less attention has been given to the experiences of family caregivers, who often bear much responsibility in caring for and managing the patient’s care during transitions across health settings. When it comes to care transitions, family caregivers need more information and knowledge about how to care for the patient, need to be more involved in identifying patient needs [8], want to feel cared for and about by medical providers, and want to feel prepared and capable of implementing patient care plans [9]. Family caregivers need information during every step of the process, both before and after hospital discharge, which underscores the importance of communication with healthcare professionals across the continuum of care [10].

Understanding patient and family caregiver experience with healthcare is critical in moving toward care that is more patient-and-family-centered. While there are numerous measurement instruments designed to assess patient experience with healthcare, only a subset of these include a focus on care coordination across the continuum of care or family caregiver experiences with care transitions from hospital to home. Therefore, the purpose of this study was to develop and administer survey instruments that assess patient and family caregiver experiences with care transitions from hospital to home and examine the psychometric properties of the surveys. The surveys were designed to ask about 1) the transitional care services that matter most to patients and their family caregivers and 2) care outcomes, including the overall quality of transitional care they received, patient self-reported health, and caregiver effort/stress.

This study was undertaken as one of the specific aims of a much larger project funded by the Patient-Centered Outcomes Research Institute (PCORI) called Project ACHIEVE (**A**chieving Patient-**C**entered Care and Optimized **H**ealth **I**n Care Transitions by **E**valuating the **V**alue of **E**vidence). The overall aims of Project ACHIEVE were to identify the transitional care services and outcomes that matter most to patients and family caregivers, and to identify which combinations of transitional care strategies, or groups of services, yield desired outcomes among a large and diverse cohort of United States (U.S.) hospitals [11, 12].

## Methods

All study procedures for our survey study were approved by the Institutional Review Boards at the University of Kentucky, Kaiser Permanente Southern California, and Westat. The study protocol was carried out in accordance with relevant guidelines and regulations.

### Survey item development

To develop the content for the surveys, we first conducted a literature review to identify the types of transitional care services or components of care that are important from both the patient and family caregiver perspectives. We reviewed articles that assessed transitional care services and expected care outcomes [13–19] We identified existing relevant surveys, including Consumer Assessment of Healthcare Providers and Systems (CAHPS) surveys [20, 21] and other validated measures, such as Patient-Reported Outcomes Measurement Information System (PROMIS®) measures [22–24]. We also reviewed research and surveys on family caregiver burden and stress [25–27]. Because most existing surveys focused on either patients or caregivers, and did not comprehensively assess the range of services provided by both hospitals and outpatient providers, we sought to develop surveys to fill these important gaps. We designed surveys to ask patients and caregivers about the transitional care components and services they each received across the continuum of care from the hospital to outpatient providers once the patient returned home. We also designed the surveys to assess outcome measures of the overall quality of transitional care, patient self-reported health, and caregiver effort/stress.

The Project ACHIEVE research team conducted background research through qualitative focus groups and individual interviews with a total of 138 patients and 110 family caregivers across the U.S. [9]. This qualitative data collection elicited descriptions of patient and caregiver experiences around care transitions and identified the outcomes that were most important from their perspectives. Survey content was also informed by site visits to hospitals included in the study to better understand the facilitators and barriers of effective care transitions [28]. In addition, a Stakeholder Advisory Group (SAG), which included patient and caregiver representatives, and Scientific Advisory Council (SAC) provided important input on survey content, item wording, and survey length throughout survey development and analysis.

Synthesizing these diverse sources of input, the ACHIEVE research team identified main content areas for inclusion in the patient and caregiver surveys. We identified content areas and critical components of care provided in the hospital and at home. These components included communication with providers; communication about medications; receiving supplies or equipment; and home visits, among other critical components of care. We then drafted survey items to assess those content areas and components of care. Some survey items were adapted from existing surveys and other items were developed to assess content areas where existing items were not available or sufficient for our measurement goals.

The patient survey was designed for patients recently discharged from the hospital to assess their experiences with care both in the hospital and once they got home. The caregiver surveys were developed in parallel with the patient survey to assess similar questions about care in the hospital and at home, but from the perspective of caregivers. The caregiver surveys were designed for the family member or friend who was identified by the patient as the person who provided the most help while the patient was in the hospital and/or once they got home. The caregiver surveys had two versions intended for caregivers at two different points in time: a Time 1 (T1) caregiver who provided most of the support during the patient’s hospitalization, and a Time 2 (T2) caregiver who was most involved with the patient’s care at home. A patient could have both a T1 and T2 caregiver, or at only one of these points in time. In addition, the T1 and T2 caregiver could be the same family member or friend, or a different one.

The research team conducted cognitive interviews with patients and caregivers to pretest the draft survey items. The goal of the cognitive interviews was to assess item comprehension, relevance, and ease of responding. Sixty-eight cognitive interviews (34 patients and 34 caregivers) were conducted to iteratively test variations of the patient and caregiver survey items. Both patients and caregivers were recruited to vary in age, gender, and race/ethnicity. Results of cognitive testing were used to refine survey items prior to data collection.

### Measures

Table 1 describes the measures included in the patient and caregiver surveys, showing the number of items in each section. The patient survey included 60 items and the caregiver surveys each included 56 items. The majority of items were similar in the patient and caregiver surveys, with wording customized as needed. However, some items were unique. For example, the patient survey included items about patient-reported overall health, whereas the caregiver surveys asked about caregiver effort/stress. The surveys also included background questions about respondent characteristics. The patient and caregiver surveys were also translated into Spanish.

**Table 1:** Patient and caregiver survey sections and numbers of items

Patient Survey (60 items)	Time 1 & Time 2 Caregiver Surveys (56 items each)	Description of Similar Item Content in the Patient Survey and Time 1/Time2 Caregiver Surveys
12 items	9 items	**Introduction** (beginning)**/Background** (end) • Questions confirming respondent eligibility (patients/caregivers). Patient/caregiver sociodemographic and other characteristics.
13 items (14 in Spanish version)	15 items (16 in Spanish version)	**In the Hospital** (Single-item measures) • Transitional care services/components received in the hospital, such as whether they were told or shown what to do at home, understood what to do, practiced, received information, felt ready for discharge (1 = Yes, definitely, 2 = Yes, somewhat, 3 = No), and had a doctor’s appointment scheduled before leaving the hospital (1 = Yes, 2 = No). • Healthcare professional communication items asking if healthcare professionals explained things in a way they could understand, cared for them as a person, and if they trusted the judgment of the healthcare professionals (1 = Yes, definitely, 2 = Yes, somewhat, 3 = No).
26 items	25 items	**Since the Patient Has Been Home** (Single-item measures) • Transitional care services/components received since the patient has been home, such as medical supplies or equipment, physical or occupational therapy, or home visits from a healthcare professional (1 = Yes, 2 = No). • If the patient took medications, if they had contact information for healthcare professionals, and if they had help managing their/ the patient’s care (1 = Yes, 2 = No). • Healthcare professional communication, if they trusted the judgment of the healthcare professionals, and received conflicting information from healthcare professionals (1 = Yes, definitely, 2 = Yes, somewhat, 3 = No).
4 items	4 items	**Overall Quality of Transitional Care** (Multi-item outcome composite measure) • Ratings of the quality of care the patient received in the hospital, at home, and from healthcare professionals (1 = Poor to 5 = Excellent), including whether healthcare professionals were there for patients/caregivers as much as they needed (0 = No, 1 = Yes, somewhat, 2 = Yes, definitely).
5 items	–	**Patient Overall Health** (Multi-item outcome composite measure) • Patient-reported items from the adult version of PROMIS® (Cella et al., 2012; Hays et al., 2009; Yu et al., 2002), including physical health, mental or emotional health, sleep (1 = Poor to 5 = Excellent), bodily pain (1 = Not at all to 5 = All the time), and ability to carry out everyday activities in the past week (1 = Not at all to 5 = Completely).
–	3 items	**Caregiver Effort/Stress** (Multi-item outcome composite measure) • Caregiver-reported amount of effort (1 = No effort to 4 = A little effort) and stress (1 = Not at all stressful to 4 = Very stressful) involved in caring for the patient since the patient has been home, and whether the effort of taking care of the patient since the hospital has changed (1 = A lot easier, 3 = About the same, 5 = A lot harder).

We developed multiple survey items to assess each of the three outcomes: the Overall Quality of Transitional Care from the patient and caregiver perspectives, Patient Overall Health, and Caregiver Effort/Stress. These outcomes were proposed as composite measures or groups of two or more survey items designed to measure an underlying construct. All other items were either single-item measures or filter questions.

### Data collection

#### Hospital recruitment

Forty-three hospitals^1^ across the U.S. were recruited into the ACHIEVE Study using a purposive sampling strategy to ensure representation of the following characteristics: 1) urbanicity; 2) safety-net; 3) critical access; 4) integrated delivery system (including Kaiser Permanente hospitals); 5) participation in alternative payment models (e.g., Accountable Care Organizations); and /or 6) participation in a formal evidence-based TC program (e.g., Project RED) or community-based transitional care program (e.g., CMS Community-based Care Transitions Program (CCTP)).

#### Patient and caregiver recruitment

Medicare beneficiaries or dual-eligible patients that were discharged from the medical or surgical units at the participating hospitals were eligible to participate. Hospital staff recruited patients and Time 1 (T1—in the hospital) family member or friend caregivers of the patient. Hospital staff approached patients before discharge to obtain HIPAA authorization, consent to be contacted to complete a mail or phone survey, and contact information for a T1 caregiver who helped them during their hospitalization, if applicable. On a weekly basis throughout the data collection field period, hospital staff provided the research team with contact information for consenting discharged patients and their T1 caregivers. Contact information for Time 2 (T2—since the patient has been home) caregivers was requested from patients that completed the patient survey. Over 44 weeks of patient and caregiver recruitment from June 2017 to April 2018, 43 hospitals recruited 17,638 patients; and 41 hospitals recruited 5031 T1 caregivers (two hospitals did not recruit T1 caregivers).

#### Patient survey administration

Patients were contacted beginning 51 days after discharge per the Centers for Medicare and Medicaid Services (CMS) guidelines to avoid conflicts with Hospital Consumer Assessment of Healthcare Providers and Systems (HCAHPS) data collection. Patient survey administration included a two-wave mail survey with phone follow-up for nonrespondents. Patient data collection was conducted over 49 weeks from August 2017 through July 2018. Patients received an initial mail survey packet which included a cover letter explaining the project, the survey, and a $5 prepaid cash incentive. The exception to the prepaid cash incentive was for patients from an integrated health system, who received a $5 promised incentive upon completion of the survey, as preferred by the system’s IRB protocol.

Seven days after the initial mailing, all patients were mailed a reminder postcard and a second survey was mailed to non-respondents. After the second survey mailing, up to five follow-up phone calls were made. A Spanish language survey and materials were mailed by request; however, patients were able to complete the phone interview in English or Spanish based on their preference. On average, the patient survey was completed and/or returned 75 days after discharge.

#### Time 1 (T1) and time 2 (T2) caregiver survey administration

Both the T1 and T2 caregiver surveys were administered only by phone, with up to five phone call attempts. Caregivers were promised a $5 incentive upon completion of the survey, and interviews were conducted in both English and Spanish. Interviewers contacted the T1 caregiver 14 to 28 days after patient discharge; on average, the T1 caregiver survey was completed 18 days after patient discharge. Data were collected from T1 caregivers from July 2017 through May 2018 (about 42 weeks).

T2 caregivers were contacted at least 51 days after patient discharge, after patients completed their survey and provided the T2 caregiver name and phone number. On average, the T2 caregiver survey was completed 85 days after patient discharge. Interviewers collected data from T2 caregivers from August 2017 through July 2018 (about 47 weeks).

#### Creating the analysis datasets

To create the analysis datasets, we combined patient mail and phone survey responses and then cleaned the combined patient survey and caregiver surveys. To include a response as “complete,” a respondent had to respond to at least 50% of the applicable-to-all questions^2^ [29].

### Analyses

Several psychometric analyses were conducted with the goal of identifying conceptually meaningful and reliable outcome composite measures in the patient and caregiver surveys. We also examined the psychometric properties of the single-item measures. Psychometric analyses included (1) item response variability and missing data patterns, (2) exploratory factor analysis, (3) internal consistency and site-level reliability, and (4) correlations among the proposed outcome composite measures and other survey items.

#### Item variability and missing data

As a first step, we examined item frequencies to evaluate the variability of responses. Items with little response variability may not be helpful in differentiating higher-scoring from lower-scoring individuals and hospitals. To assess item variability, we examined either top-box scores or percent positive scores. The top-box scores (the top, most positive responses) were calculated for yes/no items with three or fewer response options (e.g. percent of respondents who answered “Yes” for items with Yes/No response options, or percent “Yes, definitely” for items with Yes, definitely/Yes, somewhat/No response options [30]). For items with four or more response options using Likert-type scales, we calculated percent positive scores (percent of respondents  who answered using the top two most positive responses [31], e.g., percent “Very well/Moderately well or Excellent/Very good). For patient-reported bodily pain, lower frequency was considered to be positive (i.e., percent Not at all/Once in the past week) as well as for caregiver effort/stress (percent No effort/A little effort and Not at all/Somewhat stressful). To indicate low item variability, we flagged items that were extremely positive with top box scores or percent positive scores greater than 95%.

Next, we identified items with high percentages of missing data. High missingness might indicate that items are not relevant to a large portion of respondents. Sources of missing data in the patient, T1 caregiver, and T2 caregiver surveys included tailored inapplicable responses (e.g., “I already knew what to do”), valid skips (based on filter questions), and other types of missing (not answered, don’t know, or refused). Items were flagged as having high missingness if all missing responses combined (tailored inapplicable, valid skips, and all other missing) exceeded 65%.

#### Exploratory factor analysis (EFA)

We conducted EFA to examine the construct validity of the three proposed outcome composite measures in the patient, T1, and T2 caregiver surveys. A separate EFA was conducted for the patient, T1, and T2 datasets. Each EFA included all items comprising the proposed outcome composite measures in each survey: Overall Quality of Transitional Care (for the patient and caregiver surveys), Patient Overall Health (patient survey), and Caregiver Effort/Stress (caregiver surveys). We used iterated principal axis factors as the method of extraction, with varimax (orthogonal) rotation to maximize the dispersion of factor loadings within factors (i.e., the number of factor loadings close to one and close to zero). Factor loadings, or correlations between items and factors, range from − 1.00 to 1.00. In general, factor loadings with absolute values above 0.40 (which explain around 16% of the variance in the item) are considered acceptable [32].

#### Outcome composite measure internal consistency reliability and site-level reliability

Next, we examined Cronbach’s alpha (α) to determine the internal consistency reliability of the items within each of the three outcome composite measures to assess whether respondents answered the items in a similar way. Cronbach’s alpha ranges from 0 to 1, with higher alphas indicating better reliability. The minimum criterion for acceptable reliability is an alpha of 0.70 [33].

To examine the variability of the outcome composite measures and single-item measures within hospitals compared to between hospitals, we computed site-level reliability. Site-level reliability, which is directly related to the standard error of measurement, captures the extent to which responses from patients and caregivers within the same hospital are more similar to each other than they are to responses from other hospitals. In other words, site-level reliability helps to assess how well a measure differentiates hospitals. It does so by comparing between-site variability to within-site variability, while adjusting for the average number of respondents within each hospital. Similar to internal consistency reliability, values of 0.70 or higher are considered acceptable for site-level reliability [33].

#### Outcome composite measure correlations

Finally, as another indicator of construct validity, we examined individual-level Spearman’s rank-order correlations among and between the outcome composite measures and the single-item measures. Since the surveys were designed to assess different, but related aspects of transitional care in hospitals, the outcome composite measures and single-item measures should be correlated, showing a correspondence or convergence that would result in moderate or moderately high correlations (e.g., +/− 0.50 to +/− 0.80). However, correlations that are very high (e.g., +/− 0.90 to +/− 1.00) may indicate a significant amount of overlap, implying that the composite measures or items may be measuring the same or very similar concepts [34]. On the other hand, correlations that are very low, close to zero, may indicate that the composite measures or items are not related to one another, potentially measuring unrelated concepts.

#### Criteria for evaluation item performance

Items that performed poorly across multiple analyses and/or in two or more surveys were dropped from the final instruments. When considering which items to drop from the surveys, we placed most emphasis on item analysis, as items with low variability and/or a high percentage of missing data would not be very useful to hospitals looking to measure and improve care transitions. Exceptions to dropping included items that were considered conceptually important to measuring care transitions and items for which a large percentage of missing data was expected (e.g., Q11_A. *Hospital: Written information in Spanish*). Demographic/background items were excluded from psychometric analysis.

## Results

Overall response rates for the patient, T1 caregiver, and T2 caregiver surveys across the 43 participating hospitals were 57% (9450/16,573), 28% (1262/4455), and 35% (1788/5106), respectively. After data cleaning, the final analysis datasets consisted of 9282 patient responses, 1245 T1 caregiver responses, and 1749 T2 caregiver responses representing 43 hospitals. Supplemental Table 1 presents the characteristics of the 43 participating hospitals and provides the comparison of study hospitals to the 2015 American Hospital Association (AHA) registered hospitals on selected characteristics. The study hospitals were more likely than AHA hospitals to be from the Northeast and West, to be large (≥300 beds), and have nongovernment/non-for-profit ownership. In addition, the study hospitals were more likely to be large urban and teaching compared to 2019 CMS Impact hospitals (Supplemental Table 2).

Table 2 presents patient respondent characteristics. The majority of patient respondents were female (53%), White (78%), and Non-Hispanic (86%). Twenty-seven percent of patient respondents had at least a 4-year college degree. Most patients (80%) had a family member or friend who helped to take care of them at home. The most common category of informal caregiver was husband/wife (53%), followed by son/daughter (including in-laws) (27%).

**Table 2:** Patient respondent characteristics (*N* = 9282)

Patient characteristics	N	%
**Gender**		
Male	4297	47%
Female	4833	53%
**Total**	**9130**	**100%**
Missing	152	
**Education**		
Some high school or less	1299	15%
High school graduate or GED	2383	27%
Some college or 2-year degree	2791	31%
4-year college graduate	993	11%
More than 4-year college degree	1447	16%
**Total**	**8913**	**100%**
Missing	369	
**Hispanic, Latino, or Spanish origin**		
Yes	1264	14%
No	7465	86%
**Total**	**8729**	**100%**
Missing	553	
**Race**		
White	6908	78%
Black or African American	840	9%
Asian	266	3%
Native Hawaiian or Other Pacific Islander	39	< 1%
American Indian or Alaska Native	75	1%
Other	533	6%
More than one race	247	3%
**Total**	**8908**	**100%**
Missing	374	
**Patient had a family member or friend who helped take care of them at home**		
Yes	7106	80%
No	1814	20%
**Total**	**8920**	**100%**
Missing	362	
**Family member or friend’s relationship to the patient (of the 7106 who answered Yes, above)**		
Husband/Wife	3269	53%
Partner/Significant Other (includes boyfriend/girlfriend)	233	4%
Son/Daughter (includes in-laws)	1642	27%
Brother/Sister (includes in-laws)	267	4%
Father/Mother (includes in-laws)	100	2%
Grandson/Granddaughter	118	2%
Other Relative	113	2%
A Friend or Someone Else	449	7%
**Total**	**6191**	**100%**
Missing	915	

Note: Totals differ due to missing data and may not sum to 100% due to rounding

T1 and T2 caregiver respondent characteristics are presented in Table 3. The majority of both T1 and T2 caregiver respondents were female (72 and 70%, respectively). Approximately one-third both T1 and T2 caregivers had at least a 4-year college degree. Most of the caregivers were not working or were retired (64% of T1 and 70% of T2), and identified as the husband or wife of the patient (58% of T1 and 61% of T2). Approximately one-quarter of both T1 and T2 caregivers identified as sons or daughters of the patient (including in-laws). The majority of caregivers had been caring for the patient for 12 months or more (58% of T1 and 56% of T2), lived with the patient (78% of T1 and 84% of T2), and identified as the patient’s sole caregiver (51% of T1 and 60% of T2).

**Table 3:** T1 (*N* = 1245) and T2 (*N* = 1749) caregiver respondent characteristics

Caregiver Characteristics	T1		T2	
	N	%	N	%
**Gender**				
Male	348	28%	527	30%
Female	874	72%	1212	70%
**Total**	**1222**	**100%**	**1739**	**100%**
Missing	23		10	
**Education**				
Some high school or less	112	9%	177	10%
High school graduate or GED	264	22%	427	25%
Some college or 2-year degree	410	34%	577	33%
4-year college graduate	214	18%	266	15%
More than 4-year college degree	210	17%	280	16%
**Total**	**1210**	**100%**	**1727**	**100%**
Missing	35		22	
**Current employment status**				
Full-time for pay	296	25%	314	18%
Full-time unpaid	14	1%	24	1%
Part-time for pay	115	10%	170	10%
Part-time unpaid	9	1%	8	< 1%
Not working or Retired	773	64%	1210	70%
**Total**	**1207**	**100%**	**1726**	**100%**
Missing	38		23	
**Relationship to patient**				
Husband/Wife	717	58%	1064	61%
Partner/Significant Other (includes boyfriend/girlfriend)	39	3%	62	4%
Son/Daughter (includes in-laws)	336	27%	408	23%
Brother/Sister (includes in-laws)	50	4%	57	3%
Father/Mother (includes in-laws)	35	3%	39	2%
Grandson/Granddaughter	19	2%	18	1%
Other Relative	13	1%	20	1%
A Friend or Someone Else	36	3%	81	5%
**Total**	**1245**	**100%**	**1749**	**100%**
Missing	0		0	
**Length of time the caregiver has taken part in or overseen patient’s care**				
Less than 3 months	371	30%	281	16%
At least 3 months but less than 12 months	149	12%	488	28%
12 months or more	705	58%	964	56%
**Total**	**1225**	**100%**	**1733**	**100%**
Missing	20		16	
**Caregiver lives with patient**				
Yes	945	78%	1465	84%
No	273	22%	270	16%
**Total**	**1218**	**100%**	**1735**	**100%**
Missing	27		14	
**Other people help caregiver care for patient**				
Yes	594	49%	684	40%
No	620	51%	1046	60%
**Total**	**1214**	**100%**	**1730**	**100%**
Missing	31		19	

Note: Totals differ due to missing data and may not sum up to 100% due to rounding

### Item variability and missingness

Table 4 presents percent positive and top-box responses for all survey items identified as having low variability and/or high percentage of missing values in the patient, T1 caregiver, and/or T2 caregiver surveys. Percent positive responses, top-box responses and missingness for all items relevant to psychometric analyses (i.e., non-background, non-demographic items) are shown in Supplemental Table 3. Percent positive/top box responses ranged from 4 to 96% in the patient survey, from 1 to 96% in the T1 caregiver survey, and from 4 to 96% in the T2 caregiver survey. Two items had percent positive scores greater than 95% (Q20. *Home: How well been able to use supplies/equipment?* 96% for patients; and Q22. *Home: How well been able to take care of wound/surgical site?* 96% for T1 and T2 caregivers). Items with excessively high or low percent positive/top box scores were flagged as having low variability.

**Table 4:** Items with high percent positive/top box scores and/or a high percentage of missing data (Patients [PT], T1 caregiver [T1], and T2 caregiver [T2] surveys)

Survey Item #			% Top box/ % Positive	% Missing
Q11_A	Hospital: Written information in Spanish?	PT	81%	95%
		T1	77%	**98%**
		T2	83%	**96%**
Q14	Hospital: Reason because needed more care at home?	PT	71%	**83%**
		T1	67%	**77%**
		T2	65%	**84%**
*Dropped*	Home: Did not take medicine … Because forgot to take	PT	64%	**87%**
*survey #*	medicine? (DROPPED FROM FINAL SURVEYS)	T1	39%	**94%**
*Q19_A*		T2	59%	**91%**
*Dropped*	Home: Did not take medicine … Because could not afford?	PT	8%	**88%**
*survey #*	(DROPPED FROM FINAL SURVEYS)	T1	1%	**94%**
*Q19_B*		T2	4%	**91%**
*Dropped*	Home: Did not take medicine … Because of medicine side	PT	29%	**88%**
*survey #*	effects? (DROPPED FROM FINAL SURVEYS)	T1	28%	**94%**
*Q19_C*		T2	28%	**91%**
*Dropped*	Home: Did not take medicine … Because didn’t know	PT	6%	**88%**
*survey #*	how/when to take medicine? (DROPPED FROM FINAL	T1	8%	**94%**
*Q19_D*	SURVEYS)	T2	13%	**91%**
Q20	Home: How well been able to use supplies/equipment?^a^	PT	**96%**	37%
		T1	93%	36%
		T2	94%	25%
Q22	Home: How well been able to take care of wound/surgical	PT	94%	**69%**
	site?^a^ (DROPPED FROM FINAL CAREGIVER	T1	**96%**	**78%**
	SURVEYS, BUT KEPT IN FINAL PATIENT SURVEY)	T2	**96%**	**74%**
Q32	Home: HC prof helped manage changes or unexpected	PT	59%	43%
	problems?	T1	77%	**69%**
		T2	72%	59%

Notes: “Q” = the final patient survey item number when the item is on the patient survey only or both the patient and caregiver surveys; “CQ” = the final caregiver survey item number when the item is only on the caregiver surveys. The percent missing includes tailored inapplicable responses (e.g., “I already knew what to do”), valid skips (based on the filter questions), and other missing (not answered, didn’t know, or refused). *HC* healthcare; *OTC* over the counter; *CG* caregiver

^a^Percent positive response, the two most positive responses, is shown for this item; all other items display top box scores

The percentages shown in Table 4 combine missingness due to tailored inapplicable responses (e.g., “I already knew what to do”), valid skips, and other sources (not answered, don’t know, or refused). Seven survey items had greater than 65% missing values in the patient, T1 caregiver, and T2 caregiver surveys, indicating that the majority of respondents in all three surveys did not answer these questions. For six of the seven items (all except Q11_A [*Hospital: Written information in Spanish*]), the high missingness was due to valid skips. Three of the seven items also had very low percentages of affirmative (“yes”) responses across all three surveys (Q19_B through Q19_D, reasons patients did not take medicine as directed), indicating that these items were not applicable to most respondents. Therefore, these three items were dropped from the final surveys. Because Q19_A (forgot to take medicine) was incomplete as a standalone item and had a high percentage of missing values in all three surveys, it was also dropped from the final surveys. Finally, Q22 was dropped from the final caregiver surveys because of its low variability and high missingness. Despite excessive missingness, Q11_A (*Hospital: Written information in Spanish*) was not considered problematic because very few respondents took the survey in Spanish, so the item was retained. In summary, four items were dropped from the final patient and caregiver surveys (Q19_A through Q19_D), and one additional item was dropped from the final caregiver surveys but kept in the final patient survey (Q22).

### Exploratory factor analysis (EFA) for proposed outcome composite measures

Results of the EFA revealed that Q38 (*Home: Rate ability to take care of self/patient*) in the Overall Quality of Transitional Care outcome composite measure had a factor loading above 0.40 on two factors in the patient survey and did not load above 0.40 on either factor in the T1 caregiver survey. We therefore removed Q38 and repeated the EFA. However, given the importance of the content measured by Q38, it was retained for all subsequent analyses as a single-item measure. Results of the final EFA for the patient survey are presented in Table 5. The EFA retained two factors. All factor loadings for items on their respective composite measures were above 0.40 (range 0.51 to 0.85).

**Table 5:** Final exploratory factor analysis factor loadings for the patient survey

Outcome composite measures and items		Factor 1	Factor 2
**Overall Quality of Transitional Care**			
Q37	Hospital: Rate hospital in preparing you for taking care of self/patient at home	0.24	**0.63**
Q39	Home: Rate care from HC professionals since home	0.20	**0.85**
Q40	Overall, have HC professionals been there as much as you needed?	0.17	**0.67**
**Patient Overall Health**			
Q41	Rate physical health	**0.76**	0.26
Q42	Rate mental/emotional health	**0.69**	0.29
Q43	Rate sleep	**0.59**	0.17
Q44	Bodily pain	**0.51**	0.06
Q45	Carry out everyday physical activities	**0.58**	0.20

Notes: “Q”= the final patient survey item number when the item is on the patient survey only or both the patient and caregiver surveys. *HC* healthcare

The EFA for both the T1 and T2 caregiver surveys also retained two factors (Table 6). All factor loadings for items on their respective composite measures were above 0.40 for both T1 and T2 caregivers (range 0.60 to 0.92 and 0.65 to 0.78, respectively).

**Table 6:** Final exploratory factor analysis factor loadings for the T1 and T2 caregiver surveys

Outcome composite measures and items			Factor 1	Factor 2
**Overall Quality of Transitional Care**				
Q37	Hospital: Rate hospital in preparing you for taking care of self/patient at home	T1	**0.62**	0.17
		T2	**0.69**	0.14
Q39	Home: Rate care from HC profs since home	T1	**0.69**	0.07
		T2	**0.76**	0.07
Q40	Overall, have HC profs been there as much as you needed?	T1	**0.66**	0.10
		T2	**0.65**	0.09
**Caregiver Effort/Stress**				
CQ44	Home: How much effort for CG to care for patient?	T1	0.08	**0.60**
		T2	0.08	**0.72**
CQ45	Home: How stressful for CG to care for patient?	T1	0.18	**0.92**
		T2	0.14	**0.78**

Notes: “Q” = the final patient survey item number when the item is on the patient survey only or both the patient and caregiver surveys; “CQ” = the final caregiver survey item number when the item is only on the caregiver surveys. *HC* healthcare; *CG* caregiver

The pattern and magnitudes of the factor loadings in all three surveys indicated a clear differentiation between the factors, reflecting the proposed measurement structure.

### Outcome composite measure internal consistency reliability

Table 7 presents Cronbach’s alpha (α) measuring internal consistency reliability for each outcome composite measure, as well as alpha if an item were to be deleted. The two outcome composite measures in the patient survey—Overall Quality of Transitional Care and Patient Overall Health—had internal consistency reliability above the criterion of at least 0.70 (α = 0.79 for both composite measures). Similarly, the two outcome composite measures in the T1 and T2 caregiver surveys—Overall Quality of Transitional Care and Caregiver Effort/Stress—had internal consistency reliabilities of at least 0.70 (T1 α = 0.70, 0.73 and T2 α = 0.75, 0.72, respectively). For all three surveys, deleting any items would not improve reliability.

**Table 7:** Outcome composite measure internal consistency reliability (Patients [PT], T1 caregiver [T1], T2 caregiver [T2] surveys)

Outcome composite measures and items		Internal consistency reliability (Alpha if item deleted next to each item)		
		PT	T1	T2
**Overall Quality of Transitional Care**		**0.79**	**0.70**	**0.75**
Q37	Hospital: Rate hospital in preparing you for taking care of self/patient at home	0.75	0.63	0.67
Q39	Home: Rate care from HC profs since home	0.64	0.59	0.63
Q40	Overall, have HC profs been there as much as you needed?	0.74	0.61	0.69
**Patient Overall Health**		**0.79**	–	–
Q41	Rate physical health	0.71	–	–
Q42	Rate mental/emotional health	0.73	–	–
Q43	Rate sleep	0.76	–	–
Q44	Bodily pain	0.79	–	–
Q45	Carry out everyday physical activities	0.76	–	–
**Caregiver Effort/Stress**		–	**0.73**	**0.72**
CQ44	Home: How much effort for CG to care for patient?	–	–	–
CQ45	Home: How stressful for CG to care for patient?	–	–	–

Notes: “Q” = the final patient survey item number when the item is on the patient survey only or both the patient and caregiver surveys; “CQ” = the final caregiver survey item number when the item is only on the caregiver surveys. *HC* healthcare; *CG* caregiver

### Outcome composite measure site-level reliability

The site-level reliability for the outcome composite measures in the patient survey was 0.82 for Overall Quality of Transitional Care and 0.78 for Patient Overall Health. The site-level reliability for Overall Quality of Transitional Care was 0.72 in the T1 caregiver survey and 0.62 for the T2 caregiver survey (below the criterion of 0.70). The Caregiver Effort/Stress outcome composite measure had site-level reliability below the criterion for both T1 (0.63) and T2 (0.64) caregivers. Site-level reliability for all final survey items is shown in Supplemental Table 4.

### Outcome composite measure correlations

In both the patient and caregiver surveys, the Overall Quality of Transitional Care outcome composite measure was significantly correlated with the other outcome composite measures and most of the single-item measures. In the patient survey, higher Overall Quality of Transitional Care was significantly correlated with better self-reported Patient Overall Health (*r*_s_ = 0.40, *p* < .05). In the caregiver surveys, higher Overall Quality of Transitional Care was significantly related to lower Caregiver Effort/Stress^3^ for T1 caregivers (*r*_s_ = 0.19, *p* < .05,) and for T2 caregivers (*r*_s_ = 0.18, *p* < .05). We also examined correlations between the outcome composite measures and the single-item measures (in Supplemental Table 5). Out of 40 possible associations between the single-item measures and each of the two outcome composite measures on the patient survey, the majority were statistically significant (*p* < .05) (37 correlations with Overall Quality of Transitional Care; 39 correlations with Patient Overall Health). Out of 37 possible associations between the single-item measures and each of the two outcome composite measures on the caregiver surveys, the majority were also statistically significant (*p* < .05) (32 correlations for T1 caregivers and 30 for T2 caregivers with Overall Quality of Transitional Care; 30 correlations for T1 caregivers and 33 for T2 caregivers with Caregiver Effort/Stress).

### Final survey items

After reviewing the performance of the single-item measures, we identified and removed items with multiple analytic issues from the final patient and caregiver surveys (four items from the patient survey, five from the T1 caregiver survey, and five from the T2 caregiver survey, with dropped items shown in Supplemental Table 6). The final patient, T1 caregiver, and T2 caregiver surveys (shown in Additional Files 7, 8, and 9-- Appendixes A, B, and C) have 56, 51, and 51 items respectively.

## Discussion

Lack of appropriate, well-organized transitions can lead to unplanned hospital readmissions and poor patient outcomes. While hospitals continue to focus efforts to improve care transitions and reduce readmissions, it is important to understand the care transitions experience from the perspective of patients and their family caregivers when deciding where to invest finite resources. Our study developed and tested patient and caregiver surveys designed to assess 1) the transitional care services or components of care that are provided in the hospital and at home, and 2) care outcomes including the overall quality of transitional care they received, patient self-reported health, and caregiver effort/stress. Importantly, the transitional care services included in the survey were based on multiple sources of research and input about what matters most to patients and family caregivers in transitional care [9, 13–17].

Across the patient and caregiver surveys, most items had reasonable response variability and missingness, even though overall responses tended to be positive. Percent positive or top box scores were similar between T1 and T2 caregivers. Only four items were dropped from the final patient survey and five items were dropped from the final caregiver surveys because of low variability and/or a high percentage of missingness.

When examining the factor structure of the three proposed outcome composite measures—Overall Quality of Transitional Care (patient and caregiver surveys), Patient Overall Health (patient survey), and Caregiver Effort/Stress (caregiver surveys)—the final factor analyses yielded good item factor loadings (above 0.40) that supported the construct validity of the three final outcome composite measures. The final outcome composite measures also demonstrated good internal consistency reliability (above 0.70).

Site level reliability of the composite measures was also good for the patient survey, but did not reach acceptable levels for T2 caregivers on Overall Quality of Transitional Care, and for T1 and T2 caregivers on Caregiver Effort/Stress. Because T1 and T2 caregivers were recruited through patients and response rates were much lower for caregivers than for patients (28% for T1 and 35% for T2 caregivers), the average number of caregiver respondents associated with a specific hospital was low (for the items in the final survey the average number of caregivers ranged from 8 to 30 for T1s and 7 to 41 for T2s). Therefore, it is likely that the small number of respondents affected the site level reliability of the caregiver composite measure scores.

In the patient survey, higher Overall Quality of Transitional Care was significantly correlated with better Patient Health Outcomes, demonstrating a positive relationship between the quality of transitional care and patient-reported physical, mental/emotional health, sleep, pain and mobility. Higher Overall Quality of Transitional Care for T1 and T2 caregivers was also significantly correlated with lower Caregiver Effort/Stress, again demonstrating an important relationship among these key outcomes. In addition, the majority of the single-item measures within each survey were also related to the outcome composite measures. These significant correlations support the construct validity of the outcome composite measures in relation to the single-item measures. Including built-in “outcome” composite measures within the surveys enables analyses to examine how receiving certain transitional care services or components relates to the overall quality of transitional care, patient-reported health outcomes, and caregiver effort/stress, which are important patient and family-centered outcomes of care.

Because the survey items ask about many different types of services or components of care, including communication, providing education and information, medical supplies or equipment, transportation assistance, meals, and physical or occupational therapy, most of the items are not grouped into composite measures. Instead, the single-item measures that assess the type or number of services or components of care can be used to identify which groups or clusters of services result in more positive patient and caregiver experiences and outcomes. Another study by the Project ACHIEVE team [12] conducted analyses of survey data from hospitals and data from the patient survey and identified five groups of transitional care components or strategies that were most likely to co-occur and be delivered by hospitals. The strategies patients reported receiving were more important in predicting 30-day hospital readmissions than strategies that hospitals reported delivering, highlighting the importance of patient experience as a driver of outcomes.

Our study included a comprehensive survey development and testing process that included extensive involvement and input from patients, caregivers, and researchers throughout the entire process. In addition, our study’s large-scale data collection spanned almost an entire year with thousands of patients and caregivers. While there are numerous existing measures that assess patient experience with healthcare delivery in various settings of care, there are a limited number of measures that specifically focus on transitions of care, and even fewer that obtain a family caregiver perspective. In addition, few surveys focus on the transitional care services provided both in the hospital and in the outpatient setting after patients return home.

In a systematic review of research on the quality of transitional care interventions, Allen et al. [35] concluded that there was a need for improved understanding and evidence about the quality of transitional care for older patients and their caregivers. In particular, they called for more research on patient and caregiver experiences, caregiver burden and support, and emotional support for older patients and their caregivers during care transitions. Our survey study involving Medicare beneficiaries and dual-eligible patients and their caregivers provides much-needed measurement tools to enable future research in these areas to fill these important gaps, especially in the measurement of family caregiver experiences with care transitions [36].

The patient and caregiver surveys developed by our study can be used independently to assess patient and caregiver experiences with care transitions, but the parallel focus of surveys also allows for more nuanced comparisons. Patients can identify their family caregivers so patient and caregiver experiences on equivalent items can be compared, as well as comparisons between caregivers that provided assistance during the patient’s hospital stay and those that assisted the patient at home. The corresponding nature of the outcome composite measures and single-item measures in the patient and caregiver surveys adds new possibilities for examining associations among these critical perspectives on care transitions.

### Strengths and limitations

Our study’s strengths include broad input from a Stakeholder Advisory Group (SAG), which included patient and caregiver representatives, a Scientific Advisory Council (SAC), and the Project ACHIEVE research team, at key steps in the survey development and testing process which enabled us to incorporate comprehensive content about transitional care services received both in the hospital and at home. In addition, our iterative development process involved extensive cognitive testing prior to main data collection. Furthermore, caregivers were identified by and linked to patients rather than using unrelated samples of patients and caregivers. Finally, we conducted our study with a large sample of hospitals, patients, and caregivers over an extended period of data collection of 49 weeks.

There were also several limitations of the study. The 51-day wait time constraint before we could begin patient data collection, that was imposed to avoid overlap with Hospital CAHPS patient experience data collection, is very likely to have affected patient recall of their hospital experiences. While we tried to overcome that limitation by surveying T1 caregivers shortly after the patient’s discharge, the caregivers’ responses are not a proxy for patients since they are from a different perspective. Although we had high response rates for patients, caregiver response rates were lower and because patients did not always identify caregivers, the actual number of caregiver responses was low relative to patient responses. In addition, although we had a large sample of hospitals, it is possible that those hospitals that agreed to participate in the study may already have been more engaged in transitional care than hospitals not included in the study and therefore led to more positive survey results. Finally, the majority of patient respondents were white (78%) and high-school educated or higher (85%) so the results obtained in our study may not be as generalizable to more diverse patient populations.

## Conclusions

Psychometric analyses provided overall support for the three outcome composite measures and single-item measures in the final patient and caregiver surveys. The final patient, Time 1, and T2 caregiver surveys are psychometrically sound and can be used by health systems, hospitals and healthcare researchers to assess care transitions and related outcomes. Results from these surveys can be used to improve care transitions and outcomes, focusing on what matters most to patients and their family caregivers.

## Supplementary Information

**Additional file 1. **

**Additional file 2.**

**Additional file 3.**

**Additional file 4.**

**Additional file 5.**

**Additional file 6.**

**Additional file 7.**

**Additional file 8.**

**Additional file 9.**

## Data Availability

The datasets used and/or analyzed during the current study are available from the corresponding author on reasonable request.

## Abbreviations

ACHIEVE: Achieving patient-centered care and optimized health in care transitions by evaluating the value of evidence
AHA: American hospital association
ATA: Applicable-to-all
CAHPS: Consumer assessment of healthcare providers and systems
CCTP: Community-based care transitions program
CG: Caregiver
CMS: U.S. centers for medicare and medicaid services
EFA: Exploratory factor analysis
FFS: Fee-for-service
HC: Healthcare
HCAHPS: Hospital consumer assessment of healthcare providers and systems
IRB: Institutional review board
OTC: Over the counter
PCORI: Patient-centered outcomes research institute
PROMIS: Patient-reported outcomes measurement information system
SAC: Scientific advisory council
SAG: Stakeholder advisory group
T1: Time 1
T2: Time 2
US: United States

## Footnotes

1 The 43 hospitals include two locations of the same hospital, which were counted as separate entities for the purposes of psychometric analysis, but considered a single site in the larger Project ACHIEVE analytic study.

2 “Applicable to all” indicates if a question was applicable to all respondents, excluding questions skipped based on previous responses.

3 Caregiver Effort/Stress was reverse-coded so “No/A little effort” and “Not at all/Somewhat stressful” were higher/more positive scores.
