# Supplementary material for: Development and psychometric properties of surveys to assess patient and family caregiver experience with care transitions
Source: BMC Health Serv Res. 2021 Aug 9;21:785. doi: 10.1186/s12913-021-06766-w (PMC8353769; doi:10.1186/s12913-021-06766-w)
Supplement: Supplementary file 1 — Additional file 1. [file 12913_2021_6766_MOESM1_ESM.docx]

**Title Page**

Development and psychometric properties of surveys to assess patient and family caregiver experience with care transitions

**Authors**

Joann Sorra, PhD*

Westat, Rockville, Maryland, USA

Katarzyna Zebrak, PhD

Westat, Rockville, Maryland, USA

Deborah Carpenter, RN, MSN

Westat, (retired), Rockville, Maryland, USA

Theresa Famolaro, MPS, MS, MBA

Westat, Rockville, Maryland, USA

John Rauch

Westat (retired), Rockville, Maryland, USA

Jing Li, MD, DrPH, MS

Center for Health Services Research, University of Kentucky, Lexington, Kentucky, USA

Terry Davis, PhD

Louisiana State University Health Shreveport, Shreveport, Louisiana, USA

Huong Q. Nguyen, RN, PhD

Kaiser Permanente Southern California, Pasadena, California, USA

Megan McIntosh

Center for Health Services Research, University of Kentucky, Lexington, Kentucky, USA

Suzanne Mitchell, MD, MS

Boston Medical Center/Boston University School of Medicine, Boston, Massachusetts, USA

Karen B. Hirschman, PhD MSW
NewCourtland Center for Transitions and Health, University of Pennsylvania School of Nursing, Philadelphia, Pennsylvania, USA

Carol Levine, MA

United Hospital Fund, New York, New York, USA

Jessica Miller Clouser, MPH

Center for Health Services Research, University of Kentucky, Lexington, Kentucky, USA

Jane Brock, MD, MSPH

Telligen, Greenwood Village, Colorado, USA

Mark V. Williams, MD

Center for Health Services Research, University of Kentucky, Lexington, Kentucky, USA

* Indicates corresponding author: joannsorra@westat.com

**Supplemental Table 1. Distribution of study hospitals by AHA hospital characteristics^[[1]](#footnote-1)^**

| Hospital Characteristic | Study Hospitals  (N = 43) | | AHA Hospitals  (N = 6,251) | |
| --- | --- | --- | --- | --- |
| Geographic Region^[[2]](#footnote-2)^ | N | % | N | % |
| Midwest | 8 | 19% | 1,701 | 27% |
| Northeast | 10 | 23% | 803 | 13% |
| South | 8 | 19% | 2,576 | 41% |
| West | 17 | 40% | 1,171 | 19% |
| Licensed Beds |  |  |  |  |
| <100 | 6 | 14% | 3,452 | 55% |
| 100-299 | 14 | 33% | 1,909 | 31% |
| ≥300 | 23 | 53% | 890 | 14% |
| Ownership |  |  |  |  |
| Government, non-federal | 8 | 19% | 1,476 | 24% |
| Nongovernment, non-for-profit | 34 | 79% | 3,099 | 50% |
| Investor-owned, for-profit | 1 | 2% | 1,676 | 27% |

1. Based on the 2015 AHA Annual Survey Data Set [↑](#footnote-ref-1)
2. States and territories are categorized into the following regions:

   Midwest: IL, IN, MI, OH, WI, IA, KS, MN, MO, NE, ND, SD

   Northeast: CT, ME, MA, NH, RI, VT, NJ, NY, PA

   South: DE, FL, GA, MD, NC, SC, VA, DC, WV, AL, KY, MS, TN, AR, LA, OK, TX

   West: AZ, CO, ID, MT, NV, NM, UT, WY, CA, OR, WA [↑](#footnote-ref-2)
