# Supplementary material for: Development and psychometric properties of surveys to assess patient and family caregiver experience with care transitions
Source: BMC Health Serv Res. 2021 Aug 9;21:785. doi: 10.1186/s12913-021-06766-w (PMC8353769; doi:10.1186/s12913-021-06766-w)
Supplement: Supplementary file 3 — Additional file 3. [file 12913_2021_6766_MOESM3_ESM.docx]

**Title Page**

Development and psychometric properties of surveys to assess patient and family caregiver experience with care transitions

**Authors**

Joann Sorra, PhD*

Westat, Rockville, Maryland, USA

Katarzyna Zebrak, PhD

Westat, Rockville, Maryland, USA

Deborah Carpenter, RN, MSN

Westat, (retired), Rockville, Maryland, USA

Theresa Famolaro, MPS, MS, MBA

Westat, Rockville, Maryland, USA

John Rauch

Westat (retired), Rockville, Maryland, USA

Jing Li, MD, DrPH, MS

Center for Health Services Research, University of Kentucky, Lexington, Kentucky, USA

Terry Davis, PhD

Louisiana State University Health Shreveport, Shreveport, Louisiana, USA

Huong Q. Nguyen, RN, PhD

Kaiser Permanente Southern California, Pasadena, California, USA

Megan McIntosh

Center for Health Services Research, University of Kentucky, Lexington, Kentucky, USA

Suzanne Mitchell, MD, MS

Boston Medical Center/Boston University School of Medicine, Boston, Massachusetts, USA

Karen B. Hirschman, PhD MSW
NewCourtland Center for Transitions and Health, University of Pennsylvania School of Nursing, Philadelphia, Pennsylvania, USA

Carol Levine, MA

United Hospital Fund, New York, New York, USA

Jessica Miller Clouser, MPH

Center for Health Services Research, University of Kentucky, Lexington, Kentucky, USA

Jane Brock, MD, MSPH

Telligen, Greenwood Village, Colorado, USA

Mark V. Williams, MD

Center for Health Services Research, University of Kentucky, Lexington, Kentucky, USA

* Indicates corresponding author: joannsorra@westat.com

**Supplemental Table 3. Individual-level item variability – Percent positive/top box scores and percent of missing data (Patients [PT], T1 caregiver [T1], and T2 caregiver [T2] surveys)**

| **Survey**  **Item #** | |  | **% Top box/**  **% Positive** | **% Missing** |
| --- | --- | --- | --- | --- |
| Q2 | Hospital: Were you told/shown what to do? | PT | 72% | 10% |
|  |  | T1 | 62% | 27% |
|  |  | T2 | 69% | 43% |
| Q3 | Hospital: Understood what to do at home? | PT | 71% | 3% |
|  |  | T1 | 64% | 9% |
|  |  | T2 | 68% | 31% |
| Q4 | Hospital: Get to practice things you would need to do at | PT | 50% | 21% |
|  | home? | T1 | 46% | 53% |
|  |  | T2 | 51% | ***65%*** |
| Q5 | Hospital: Explain things in a way you could understand? | PT | 81% | 3% |
|  |  | T1 | 83% | 9% |
|  |  | T2 | 84% | 31% |
| Q6 | Hospital: Cared about you as a person? | PT | 84% | 3% |
|  |  | T1 | 73% | 9% |
|  |  | T2 | 74% | 32% |
| Q7 | Hospital: Trusted HC professionals’ judgments? | PT | 80% | 3% |
|  |  | T1 | 77% | 9% |
|  |  | T2 | 79% | 31% |
| Q8 | Hospital: Got information about symptoms to watch out | PT | 69% | 3% |
|  | for? | T1 | 67% | 9% |
|  |  | T2 | 74% | 32% |
| Q9 | Hospital: HC professional talked to you about prescription | PT | 76% | 6% |
|  | and OTC medicines? (filter question) | T1 | 77% | 12% |
|  |  | T2 | 76% | 33% |
| Q10 | Hospital: Were side effects of medicine clear? | PT | 61% | 15% |
|  |  | T1 | 62% | 25% |
|  |  | T2 | 63% | 42% |
| Q11 | Hospital: Helpfulness of written information^a^ | PT | 90% | 11% |
|  |  | T1 | 93% | 30% |
|  |  | T2 | 92% | 42% |
| Q11_A | Hospital: Written information in Spanish? | PT | 81% | **95%** |
|  |  | T1 | 77% | **98%** |
|  |  | T2 | 83% | **96%** |
| Q12 | Hospital: Was doctor appointment scheduled? | PT | 85% | 2% |
|  |  | T1 | 85% | 12% |
|  |  | T2 | 89% | 33% |
| Q13(R) | Hospital: Was it too soon to leave hospital? (negatively | PT | 82% | 2% |
|  | worded, reverse coded) (filter question) | T1 | 73% | 10% |
|  |  | T2 | 77% | 31% |
| Q14 | Hospital: Reason because needed more care at home? | PT | 71% | **83%** |
|  |  | T1 | 67% | **77%** |
|  |  | T2 | 65% | **84%** |
| Q15 | Home: Had HC prof contact info? (filter question) | PT | 88% | 2% |
|  |  | T1 | 88% | 16% |
|  |  | T2 | 86% | 2% |
| Q16 | Home: Got help with problems or questions [when you | PT | 80% | 42% |
|  | contacted HC professionals]? | T1 | 82% | 57% |
|  |  | T2 | 82% | 45% |
| Q17 | Home: Had to take any prescription or OTC medicine? | PT | 88% | 2% |
|  | (filter question) | T1 | ***95%*** | 16% |
|  |  | T2 | 93% | 1% |
| Q18(R) | Home: Has there been a time when did not take medicine | PT | 84% | 17% |
|  | As directed? (negatively worded, reverse coded) (filter | T1 | 92% | 21% |
|  | question) | T2 | 90% | 10% |
| *Dropped* | Home: Did not take medicine… Because forgot to take | PT | 64% | **87%** |
| *survey #* | medicine? (DROPPED FROM FINAL SURVEYS) | T1 | 39% | **94%** |
| *Q19_A* |  | T2 | 59% | **91%** |
| *Dropped* | Home: Did not take medicine… Because could not afford? | PT | 8% | **88%** |
| *survey #* | (DROPPED FROM FINAL SURVEYS) | T1 | 1% | **94%** |
| *Q19_B* |  | T2 | 4% | **91%** |
| *Dropped* | Home: Did not take medicine… Because of medicine side | PT | 29% | **88%** |
| *survey #* | effects? (DROPPED FROM FINAL SURVEYS) | T1 | 28% | **94%** |
| *Q19_C* |  | T2 | 28% | **91%** |
| *Dropped* | Home: Did not take medicine… Because didn't know | PT | 6% | **88%** |
| *survey #* | how/when to take medicine? (DROPPED FROM FINAL | T1 | 8% | **94%** |
| *Q19_D* | SURVEYS) | T2 | 13% | **91%** |
| Q19 | Home: Needed to use supplies or equipment? (filter | PT | 67% | 2% |
|  | question) | T1 | 78% | 16% |
|  |  | T2 | 76% | <1% |
| Q20 | Home: How well been able to use supplies/equipment?^a^ | PT | **96%** | 37% |
|  |  | T1 | 93% | 36% |
|  |  | T2 | 94% | 25% |
| Q21 | Home: Had to take care of wound or surgical site? (filter | PT | 32% | 3% |
|  | question) | T1 | 27% | 16% |
|  |  | T2 | 26% | 1% |
| Q22 | Home: How well been able to take care of wound/surgical | PT | 94% | **69%** |
|  | site?^a^ (DROPPED FROM FINAL CAREGIVER | T1 | **96%** | **78%** |
|  | SURVEYS, BUT KEPT IN FINAL PATIENT SURVEY) | T2 | **96%** | **74%** |
| Q23 | Home: Received transportation assistance? (filter question) | PT | 10% | 2% |
|  |  | T1 | --- | --- |
|  |  | T2 | --- | --- |
| Q24 | Home: Wanted transportation assistance? | PT | 11% | 21% |
|  |  | T1 | --- | --- |
|  |  | T2 | --- | --- |
| Q25 | Home: Received meals? (filter question) | PT | 4% | 1% |
|  |  | T1 | --- | --- |
|  |  | T2 | --- | --- |
| Q26 | Home: Wanted meals? | PT | 9% | 13% |
|  |  | T1 | --- | --- |
|  |  | T2 | --- | --- |
| Q27 | Home: Received physical/occupational therapy? (filter | PT | 44% | 2% |
|  | question) | T1 | --- | --- |
|  |  | T2 | --- | --- |
| Q28 | Home: Needed physical/occupational therapy? | PT | 10% | 49% |
|  |  | T1 | --- | --- |
|  |  | T2 | --- | --- |
| Q29 | Home: Had home visit? (filter question) | PT | 49% | 2% |
|  |  | T1 | 52% | 17% |
|  |  | T2 | 52% | 1% |
| Q30 | Home: Wanted home visit? | PT | 6% | 52% |
|  |  | T1 | 14% | 61% |
|  |  | T2 | 8% | 53% |
| Q31 | Home: Talked with HC professional? (filter question) | PT | 85% | 2% |
|  |  | T1 | 67% | 16% |
|  |  | T2 | 67% | 1% |
| Q32 | Home: HC prof helped manage changes or unexpected | PT | 59% | 43% |
|  | problems? | T1 | 77% | **69%** |
|  |  | T2 | 72% | 59% |
| Q33 | Home: Explained things in a way you could understand? | PT | 82% | 20% |
|  |  | T1 | 89% | 45% |
|  |  | T2 | 88% | 34% |
| Q34 | Home: Cared about you as a person? | PT | 85% | 20% |
|  |  | T1 | 75% | 46% |
|  |  | T2 | 71% | 35% |
| Q35 | Home: Trusted HC prof's judgments? | PT | 81% | 20% |
|  |  | T1 | 83% | 45% |
|  |  | T2 | 81% | 34% |
| Q36(R) | Home: HC professional told you something that went | PT | 87% | 20% |
|  | against what another HC professional said? (negatively | T1 | 84% | 45% |
|  | worded) (reversed) | T2 | 83% | 34% |
| Q37 | Hospital: Rate hospital in preparing you for taking care of | PT | 79% | 4% |
|  | self/patient at home^a^ | T1 | 76% | 13% |
|  |  | T2 | 79% | 32% |
| Q38 | Home: Rate ability to take care of self/patient^a^ | PT | 72% | 3% |
|  |  | T1 | 85% | 17% |
|  |  | T2 | 85% | 1% |
| Q39 | Home: Rate care from HC profs since home^a^ | PT | 82% | 16% |
|  |  | T1 | 86% | 32% |
|  |  | T2 | 85% | 10% |
| Q40 | Overall, have HC profs been there as much as you | PT | 72% | 5% |
|  | needed? | T1 | 70% | 5% |
|  |  | T2 | 73% | 3% |
| Q41 | Rate physical health^a^ | PT | 43% | 3% |
|  |  | T1 | --- | --- |
|  |  | T2 | --- | --- |
| Q42 | Rate mental/emotional health^a^ | PT | 61% | 3% |
|  |  | T1 | --- | --- |
|  |  | T2 | --- | --- |
| Q43 | Rate sleep^a^ | PT | 38% | 3% |
|  |  | T1 | --- | --- |
|  |  | T2 | --- | --- |
| Q44 | Bodily pain^a^ | PT | 40% | 4% |
|  |  | T1 | --- | --- |
|  |  | T2 | --- | --- |
| Q45 | Carry out everyday physical activities^a^ | PT | 49% | 3% |
|  |  | T1 | --- | --- |
|  |  | T2 | --- | --- |
| Q46 | Home: Has a family member or friend helped care for | PT | 80% | 4% |
|  | you? | T1 | --- | --- |
|  |  | T2 | --- | --- |
| Q49 | How confident are you in filling out medical forms by | PT | 75% | 2% |
|  | yourself?^a^ | T1 | --- | --- |
|  |  | T2 | --- | --- |
| Q50 | Do you usually ask someone to help you read materials | PT | 25% | 2% |
|  | you receive from the hospital? | T1 | --- | --- |
|  |  | T2 | --- | --- |
| CQ5 | Hospital: Did CG talk with any HC in hospital about the | PT | --- | --- |
|  | patient? (filter question) | T1 | 92% | <1% |
|  |  | T2 | 86% | 19% |
| CQ15 | Hospital: Did CG receive written information? (filter | PT | --- | --- |
|  | question) | T1 | 87% | 14% |
|  |  | T2 | 93% | 34% |
| CQ29 | Home: Patient received help for mental health problem? | PT | --- | --- |
|  | (filter question) | T1 | 12% | 17% |
|  |  | T2 | 14% | 2% |
| CQ30 | Home: CG wanted patient to receive help for mental | PT | --- | --- |
|  | health problem? | T1 | 91% | 29% |
|  |  | T2 | 92% | 18% |
| CQ33 | Home visit scheduled when caregiver could be present?^a^ | PT | --- | --- |
|  |  | T1 | 82% | 57% |
|  |  | T2 | 80% | 50% |
| CQ44 | Home: How much effort for CG to care for patient?^a^ | PT | --- | --- |
|  |  | T1 | 36% | 17% |
|  |  | T2 | 40% | 1% |
| CQ45 | Home: How stressful for CG to care for patient?^a^ | PT | --- | --- |
|  |  | T1 | 68% | 17% |
|  |  | T2 | 66% | 1% |
| CQ46 | How caring for patient has changed from hospital until | PT | --- | --- |
|  | now?^a^ | T1 | 51% | 17% |
|  |  | T2 | 58% | 2% |

Notes: “Q”= the final patient survey item number when the item is on the patient survey only or both the patient and caregiver surveys; “CQ” = the final caregiver survey item number when the item is on the caregiver survey only. The percent missing includes tailored inapplicable responses (e.g., “I already knew what to do”), valid skips (based on the filter questions), and other missing (not answered, didn’t know, or refused). HC = healthcare; OTC = over the counter; CG = caregiver.

^a^Percent positive response, the two most positive responses, is shown for this item; all other items display top box scores.
