# Supplementary material for: Development and psychometric properties of surveys to assess patient and family caregiver experience with care transitions
Source: BMC Health Serv Res. 2021 Aug 9;21:785. doi: 10.1186/s12913-021-06766-w (PMC8353769; doi:10.1186/s12913-021-06766-w)
Supplement: Supplementary file 5 — Additional file 5. [file 12913_2021_6766_MOESM5_ESM.docx]

**Title Page**

Development and psychometric properties of surveys to assess patient and family caregiver experience with care transitions

**Authors**

Joann Sorra, PhD*

Westat, Rockville, Maryland, USA

Katarzyna Zebrak, PhD

Westat, Rockville, Maryland, USA

Deborah Carpenter, RN, MSN

Westat, (retired), Rockville, Maryland, USA

Theresa Famolaro, MPS, MS, MBA

Westat, Rockville, Maryland, USA

John Rauch

Westat (retired), Rockville, Maryland, USA

Jing Li, MD, DrPH, MS

Center for Health Services Research, University of Kentucky, Lexington, Kentucky, USA

Terry Davis, PhD

Louisiana State University Health Shreveport, Shreveport, Louisiana, USA

Huong Q. Nguyen, RN, PhD

Kaiser Permanente Southern California, Pasadena, California, USA

Megan McIntosh

Center for Health Services Research, University of Kentucky, Lexington, Kentucky, USA

Suzanne Mitchell, MD, MS

Boston Medical Center/Boston University School of Medicine, Boston, Massachusetts, USA

Karen B. Hirschman, PhD MSW
NewCourtland Center for Transitions and Health, University of Pennsylvania School of Nursing, Philadelphia, Pennsylvania, USA

Carol Levine, MA

United Hospital Fund, New York, New York, USA

Jessica Miller Clouser, MPH

Center for Health Services Research, University of Kentucky, Lexington, Kentucky, USA

Jane Brock, MD, MSPH

Telligen, Greenwood Village, Colorado, USA

Mark V. Williams, MD

Center for Health Services Research, University of Kentucky, Lexington, Kentucky, USA

* Indicates corresponding author: joannsorra@westat.com

**Supplemental Table 5. Individual–level correlations between survey items and composite measures (Patients [PT], Time 1 caregivers [T1], Time 2 caregivers [T2])**

| **Survey items** | |  | **Overall Quality of Transitional Care** | **Patient Overall Health** | **Caregiver Effort/Stress** |
| --- | --- | --- | --- | --- | --- |
| Q2 | Hospital: Were you told/shown what to do? | PT | **0.50** | **0.22** | --- |
|  |  | T1 | **0.49** | --- | 0.05 |
|  |  | T2 | **0.45** | --- | **0.16** |
| Q3 | Hospital: Understood what to do at home? | PT | **0.48** | **0.20** | --- |
|  |  | T1 | **0.45** | --- | 0.04 |
|  |  | T2 | **0.45** | --- | **0.16** |
| Q4 | Hospital: Get to practice things you'd need to do at home? | PT | **0.44** | **0.21** | --- |
|  |  | T1 | **0.43** | --- | **0.15** |
|  |  | T2 | **0.46** | --- | **0.16** |
| Q5 | Hospital: Explain things in a way you could understand? | PT | **0.46** | **0.23** | --- |
|  |  | T1 | **0.38** | --- | **0.07** |
|  |  | T2 | **0.40** | --- | **0.15** |
| Q6 | Hospital: Cared about you as a person? | PT | **0.43** | **0.22** | --- |
|  |  | T1 | **0.45** | --- | **0.12** |
|  |  | T2 | **0.43** | --- | **0.12** |
| Q7 | Hospital: Trusted hc prof's judgments? | PT | **0.43** | **0.22** | --- |
|  |  | T1 | **0.43** | --- | **0.15** |
|  |  | T2 | **0.42** | --- | **0.14** |
| Q8 | Hospital: Got information about symptoms to watch out for? | PT | **0.45** | **0.18** | --- |
|  |  | T1 | **0.44** | --- | **0.08** |
|  |  | T2 | **0.39** | --- | **0.15** |
| Q9 | Hospital: HC prof talked to you about prescription and OTC medicines? | PT | **0.41** | **0.18** | --- |
|  |  | T1 | **0.31** | --- | -0.03 |
|  |  | T2 | **0.35** | --- | **0.07** |
| Q10 | Hospital: Were side effects of medicine clear? | PT | **0.40** | **0.20** | --- |
|  |  | T1 | **0.29** | --- | **0.10** |
|  |  | T2 | **0.35** | --- | **0.13** |
| Q11 | Hospital: Helpfulness of written information | PT | **0.47** | **0.23** | --- |
|  |  | T1 | **0.44** | --- | **0.15** |
|  |  | T2 | **0.40** | --- | **0.16** |
| Q11_A | Hospital: Written information in Spanish? | PT | **0.20** | 0.07 | --- |
|  |  | T1 | -0.18 | --- | **0.38** |
|  |  | T2 | 0.07 | --- | 0.05 |
| Q12 | Hospital: Was doctor appointment scheduled? | PT | **0.17** | **0.09** | --- |
|  |  | T1 | **0.15** | --- | -0.03 |
|  |  | T2 | **0.13** | --- | **0.13** |
| Q13 (R) | Hospital: Was it too soon to leave hospital? (negatively worded) (reversed) | PT | **0.26** | **0.22** | --- |
|  |  | T1 | **0.30** | --- | **0.17** |
|  |  | T2 | **0.28** | --- | **0.22** |
| Q14 | Hospital: Reason because needed more care at home? | PT | **-0.15** | **-0.18** | --- |
|  |  | T1 | **-0.15** | --- | **-0.24** |
|  |  | T2 | -0.08 | --- | **-0.13** |
| Q15 | Home: Had hc prof contact info? | PT | **0.22** | **0.07** | --- |
|  |  | T1 | **0.28** | --- | -0.06 |
|  |  | T2 | **0.23** | --- | **-0.08** |
| Q16 | Home: Got help with problems or questions [when you contacted hc profs]? | PT | **0.44** | **0.22** | --- |
|  |  | T1 | **0.45** | --- | **0.18** |
|  |  | T2 | **0.44** | --- | **0.11** |
| Q17 | Home: Had to take any prescription or OTC medicine? | PT | 0.02 | **-0.06** | --- |
|  |  | T1 | 0.03 | --- | **-0.11** |
|  |  | T2 | 0.01 | --- | **-0.14** |
| Q18 (R) | Home: Has there been a time when did not take medicine as directed? (negatively worded) (reversed) | PT | **0.09** | **0.11** | --- |
|  |  | T1 | **0.09** | --- | **0.13** |
|  |  | T2 | **0.06** | --- | **0.11** |
| Q19 | Home: Needed to use supplies or equipment? | PT | **-0.04** | **-0.20** | --- |
|  |  | T1 | 0.00 | --- | **-0.24** |
|  |  | T2 | -0.02 | --- | **-0.26** |
| Q20 | Home: How well been able to use supplies/equipment? | PT | **0.39** | **0.31** | --- |
|  |  | T1 | **0.26** | --- | **0.23** |
|  |  | T2 | **0.25** | --- | **0.24** |
| Q21 | Home: Had to take care of wound or surgical site? | PT | **0.03** | **-0.03** | --- |
|  |  | T1 | 0.05 | --- | **-0.15** |
|  |  | T2 | 0.01 | --- | **-0.18** |
| Q22 | Home: How well been able to take care of wound/surgical site? (DROPPED FROM FINAL CAREGIVER SURVEYS) | PT | **0.41** | **0.32** | --- |
|  |  | T1 | **0.18** | --- | **0.19** |
|  |  | T2 | **0.31** | --- | **0.17** |
| Q23 | Home: Received transportation assistance? | PT | **-0.05** | **-0.11** | --- |
|  |  | T1 | --- | --- | --- |
|  |  | T2 | --- | --- | --- |
| Q24 | Home: Wanted transportation assistance? | PT | **-0.19** | **-0.22** | --- |
|  |  | T1 | --- | --- | --- |
|  |  | T2 | --- | --- | --- |
| Q25 | Home: Received meals? | PT | **-0.04** | **-0.05** | --- |
|  |  | T1 | --- | --- | --- |
|  |  | T2 | --- | --- | --- |
| Q26 | Home: Wanted meals? | PT | **-0.16** | **-0.21** | --- |
|  |  | T1 | --- | --- | --- |
|  |  | T2 | --- | --- | --- |
| Q27 | Home: Received physical/occupational therapy? (reversed) | PT | **0.03** | **-0.08** | --- |
|  |  | T1 | --- | --- | --- |
|  |  | T2 | --- | --- | --- |
| Q28 | Home: Needed physical/occupational therapy? (reversed) | PT | **-0.20** | **-0.23** | --- |
|  |  | T1 | --- | --- | --- |
|  |  | T2 | --- | --- | --- |
| Q29 | Home: Had home visit? | PT | 0.01 | **-0.11** | --- |
|  |  | T1 | **0.07** | --- | **-0.20** |
|  |  | T2 | -0.04 | --- | **-0.22** |
| Q30 | Home: Wanted home visit? | PT | **-0.22** | **-0.20** | --- |
|  |  | T1 | **-0.29** | --- | **-0.21** |
|  |  | T2 | **-0.20** | --- | **-0.19** |
| Q31 | Home: Talked with HC professional? | PT | **0.09** | **-0.05** | --- |
|  |  | T1 | **0.14** | --- | **-0.29** |
|  |  | T2 | **0.11** | --- | **-0.23** |
| Q32 | Home: HC prof helped manage changes or unexpected problems? | PT | **0.39** | **0.13** | --- |
|  |  | T1 | **0.43** | --- | 0.07 |
|  |  | T2 | **0.43** | --- | -0.03 |
| Q33 | Home: Explained things in a way you could understand? | PT | **0.45** | **0.20** | --- |
|  |  | T1 | **0.37** | --- | **0.16** |
|  |  | T2 | **0.33** | --- | 0.03 |
| Q34 | Home: Cared about you as a person? | PT | **0.47** | **0.21** | --- |
|  |  | T1 | **0.47** | --- | **0.14** |
|  |  | T2 | **0.44** | --- | **0.12** |
| Q35 | Home: Trusted hc prof's judgments? | PT | **0.49** | **0.24** | --- |
|  |  | T1 | **0.45** | --- | **0.19** |
|  |  | T2 | **0.46** | --- | **0.14** |
| Q36 (R) | Home: HC prof told you something that went against what another hc prof said? (negatively worded) (reversed) | PT | **0.21** | **0.15** | --- |
|  |  | T1 | **0.21** | --- | **0.15** |
|  |  | T2 | **0.18** | --- | **0.21** |
| Q38 | Home: Rate ability to take care of self/patient | PT | **0.52** | **0.56** | **---** |
|  |  | T1 | **0.43** | --- | **0.21** |
|  |  | T2 | **0.46** | --- | **0.21** |
| Q46 | Home: Has a family member or friend helped care for you? | PT | -0.01 | **-0.13** | --- |
|  |  | T1 | --- | --- | --- |
|  |  | T2 | --- | --- | --- |
| Q49 | How confident are you in filling out medical forms by yourself? | PT | **0.23** | **0.33** | --- |
|  |  | T1 | --- | --- | --- |
|  |  | T2 | --- | --- | --- |
| Q50 | Do you usually ask someone to help you read materials you receive from the hospital? | PT | **-0.08** | **-0.20** | --- |
|  |  | T1 | --- | --- | --- |
|  |  | T2 | --- | --- | --- |
| CQ5 | Hospital: Did CG talk with any hc in hospital about the patient? | PT | --- | --- | --- |
|  |  | T1 | **0.07** | --- | **-0.08** |
|  |  | T2 | **0.11** | --- | **-0.15** |
| CQ15 | Hospital: Did CG receive written information? | PT | --- | --- | --- |
|  |  | T1 | **0.28** | --- | **0.08** |
|  |  | T2 | **0.21** | --- | **0.15** |
| CQ29 | Home: Patient received help for mental health problem? | PT | --- | --- | --- |
|  |  | T1 | 0.02 | --- | **-0.15** |
|  |  | T2 | -0.02 | --- | **-0.18** |
| CQ30 | Home: CG wanted patient to receive help for mental health problem? | PT | --- | --- | --- |
|  |  | T1 | **-0.18** | --- | **-0.20** |
|  |  | T2 | **-0.18** | --- | **-0.22** |
| CQ33 | Home visit scheduled when caregiver could be present? | PT | --- | --- | --- |
|  |  | T1 | **0.18** | --- | 0.02 |
|  |  | T2 | **0.20** | --- | -0.04 |
| CQ46 | How caring for patient has changed from hospital until now? | PT | --- | --- | --- |
|  |  | T1 | **0.23** | --- | **0.21** |
|  |  | T2 | **0.14** | --- | **0.19** |

^✝^Composite mean was calculated on items that were rescaled to be comparable and contribute equally to the overall composite score.

Notes: Statistically significant correlations (p < 0.05) are bolded. “Q”= the final patient survey item number when the item is on the patient survey only or both the patient and caregiver surveys; “CQ” = the final caregiver survey item number when the item is on the caregiver survey only. HC = healthcare; OTC = over the counter; CG = caregiver. Caregiver Effort/Stress was reverse-coded so “No/A little effort” and “Not at all/Somewhat stressful” were higher/more positive scores.
