# Supplementary material for: Development and psychometric properties of surveys to assess patient and family caregiver experience with care transitions
Source: BMC Health Serv Res. 2021 Aug 9;21:785. doi: 10.1186/s12913-021-06766-w (PMC8353769; doi:10.1186/s12913-021-06766-w)
Supplement: Supplementary file 6 — Additional file 6. [file 12913_2021_6766_MOESM6_ESM.docx]

**Title Page**

Development and psychometric properties of surveys to assess patient and family caregiver experience with care transitions

**Authors**

Joann Sorra, PhD*

Westat, Rockville, Maryland, USA

Katarzyna Zebrak, PhD

Westat, Rockville, Maryland, USA

Deborah Carpenter, RN, MSN

Westat, (retired), Rockville, Maryland, USA

Theresa Famolaro, MPS, MS, MBA

Westat, Rockville, Maryland, USA

John Rauch

Westat (retired), Rockville, Maryland, USA

Jing Li, MD, DrPH, MS

Center for Health Services Research, University of Kentucky, Lexington, Kentucky, USA

Terry Davis, PhD

Louisiana State University Health Shreveport, Shreveport, Louisiana, USA

Huong Q. Nguyen, RN, PhD

Kaiser Permanente Southern California, Pasadena, California, USA

Megan McIntosh

Center for Health Services Research, University of Kentucky, Lexington, Kentucky, USA

Suzanne Mitchell, MD, MS

Boston Medical Center/Boston University School of Medicine, Boston, Massachusetts, USA

Karen B. Hirschman, PhD MSW
NewCourtland Center for Transitions and Health, University of Pennsylvania School of Nursing, Philadelphia, Pennsylvania, USA

Carol Levine, MA

United Hospital Fund, New York, New York, USA

Jessica Miller Clouser, MPH

Center for Health Services Research, University of Kentucky, Lexington, Kentucky, USA

Jane Brock, MD, MSPH

Telligen, Greenwood Village, Colorado, USA

Mark V. Williams, MD

Center for Health Services Research, University of Kentucky, Lexington, Kentucky, USA

* Indicates corresponding author: joannsorra@westat.com

**Supplemental Table 6. Survey items dropped from final surveys and reason for dropping**

| **Survey Item Dropped** | | **Dropped From:** | | | **Reason(s) for dropping** |
| --- | --- | --- | --- | --- | --- |
|  |  | **Patient** | **T1 CG** | **T2 CG** |  |
| Q19A | Home: Did not take medicine… Because forgot to take medicine? | **√** | **√** | **√** | High missingness (> 65%) in all three surveys  Conceptually, not a stand-alone item |
| Q19B | Home: Did not take medicine… Because could not afford? | **√** | **√** | **√** | Very low percentage of affirmative (“yes”) responses and high missingness (> 65%) in all three surveys |
| Q19C | Home: Did not take medicine… Because of medicine side effects? | **√** | **√** | **√** | Very low percentage of affirmative (“yes”) responses and high missingness (> 65%) in all three surveys |
| Q19D | Home: Did not take medicine… Because didn't know how/when to take medicine? | **√** | **√** | **√** | Very low number of affirmative (“yes”) responses and high missingness (> 65%) in all three surveys |
| Q23 | Home: How well been able to take care of wound/surgical site? |  | **√** | **√** | Low variability and high missingness (> 65%) in caregivers surveys |
