# Supplementary material for: Development and psychometric properties of surveys to assess patient and family caregiver experience with care transitions
Source: BMC Health Serv Res. 2021 Aug 9;21:785. doi: 10.1186/s12913-021-06766-w (PMC8353769; doi:10.1186/s12913-021-06766-w)
Supplement: Supplementary file 9 — Additional file 9. [file 12913_2021_6766_MOESM9_ESM.docx]

**Title Page**

Development and psychometric properties of surveys to assess patient and family caregiver experience with care transitions

**Authors**

Joann Sorra, PhD*

Westat, Rockville, Maryland, USA

Katarzyna Zebrak, PhD

Westat, Rockville, Maryland, USA

Deborah Carpenter, RN, MSN

Westat, (retired), Rockville, Maryland, USA

Theresa Famolaro, MPS, MS, MBA

Westat, Rockville, Maryland, USA

John Rauch

Westat (retired), Rockville, Maryland, USA

Jing Li, MD, DrPH, MS

Center for Health Services Research, University of Kentucky, Lexington, Kentucky, USA

Terry Davis, PhD

Louisiana State University Health Shreveport, Shreveport, Louisiana, USA

Huong Q. Nguyen, RN, PhD

Kaiser Permanente Southern California, Pasadena, California, USA

Megan McIntosh

Center for Health Services Research, University of Kentucky, Lexington, Kentucky, USA

Suzanne Mitchell, MD, MS

Boston Medical Center/Boston University School of Medicine, Boston, Massachusetts, USA

Karen B. Hirschman, PhD MSW
NewCourtland Center for Transitions and Health, University of Pennsylvania School of Nursing, Philadelphia, Pennsylvania, USA

Carol Levine, MA

United Hospital Fund, New York, New York, USA

Jessica Miller Clouser, MPH

Center for Health Services Research, University of Kentucky, Lexington, Kentucky, USA

Jane Brock, MD, MSPH

Telligen, Greenwood Village, Colorado, USA

Mark V. Williams, MD

Center for Health Services Research, University of Kentucky, Lexington, Kentucky, USA

* Indicates corresponding author: joannsorra@westat.com

Supplemental File 9, Appendix C:

Final Time 2 Caregiver (T2) Survey

Telephone Script

Development and psychometric properties of surveys to assess patient and family caregiver experience with care transitions

This survey was developed as part of a research study funded through a Patient-Centered Outcomes Research Institute® (PCORI®) Award (TC-1403-14049).

**SURVEY NOTES**

- - **Time 2 Caregiver: (T2):** This survey is for family members or friends who helped the patient while in the hospital and/or at home after discharge. The T2 Caregiver is identified by the patient through a completed patient survey (either mail or phone) and the T2 Caregiver’s contact information is provided by the patient.
  - All questions have unread “Don’t Know” and “Refused to Answer” response options.
  - The Time 2 Caregiver (T2) survey is dependent on whether or not a Time 1 Caregiver (T1) survey was completed. For the T2 survey, there are three scenarios, Time 2 A, Time 2 B, and Time 2 C. The table below describes when each scenario should be used. Each survey item throughout the T2 Caregiver survey provided in this document contains details about that question’s scenario applicability and any wording changes for a specific scenario.

| **Scenario** | **What Happens** | **When This Survey Is Given** | **Wording Differences from Time 1 Caregiver (T1) Survey** |
| --- | --- | --- | --- |
| TIME 2 A | Hospital and home questions are asked (excluding Q20 and Q21) | When there is no T1 survey completed     OR  When T1 and T2 are **different** people and T1 did not help in the hospital | Minor Q1 differences:   - “…mentioned by [PATIENT NAME] as a person who helped them *after they left* [HOSPITAL NAME]” - “…experiences helping [PATIENT NAME] in the hospital and*/or* at home” |
| TIME 2 B | Only home questions are asked (excluding Q20 and Q21) | When T1 and T2 are **different** people and the T1 did help in the hospital | Minor Q1 differences:   - “…mentioned by [PATIENT NAME] as a person who helped them *after they left* [HOSPITAL NAME]” - “…experiences helping [PATIENT NAME] *at home*” |
| TIME 2 C | Only home questions are asked (excluding Q20 and Q21) | When T1 and T2 are the **same** person, regardless of whether or not they indicated they helped in the hospital at T1 | Minor Q1 difference:   - “…mentioned by [PATIENT NAME] as a person who helped them *after they left* [HOSPITAL NAME]”   Major Q22-Q46 wording changes:   - “Since we last talked with you” instead of “Since your [RELATIONSHIP] has been home” |

**INITIATING CONTACT**

Hello, may I please speak to [CAREGIVER NAME]?

<1> YES [GO TO INTRODUCTION]

<2> NO, NOT AVAILABLE RIGHT NOW [SET CALLBACK]

IF ASKED WHO IS CALLING:

- This is [INTERVIEWER NAME] calling from [INSERT DATA COLLECTION COMPANY] on behalf of [INSERT SPONSORING ORGANIZATION].

IF THE CAREGIVER IS NOT AVAILABLE:

- Is there a day or time that’s best to reach them?

IF THE CAREGIVER SAYS THIS IS NOT A GOOD TIME:

- If you don’t have the time now, is there a convenient day or time to call you back?

**INTRODUCTION**

*[INSERT INTRODUCTION LANGUAGE ABOUT PROJECT WHICH INCLUDES Q1]*

| [TIME 2A 2B 2C]  You were mentioned by [PATIENT’S FIRST AND LAST NAME] as a person who helped them after they left [HOSPITAL NAME] around [DATE]. Is that correct?  🞎 Yes [I would like to ask you about your experiences helping [PATIENT FIRST AND LAST NAME] in [*the hospital and/or at home*]. [CONTINUE TO INFORMED CONSENT]  🞎 No [END SURVEY, Ok. Unfortunately, you are not eligible to further participate in this survey. Thank you for your time, and have a nice [day/evening].] |
| --- |

*[INSERT INFORMED CONSENT]*

## **BACKGROUND QUESTIONS**

| 1. What is [PATIENT’S FIRST AND LAST NAME]’s relationship to you?   [INTERVIEWER SHOULD CODE IN TERMS OF WHO THE PATIENT IS IN RELATION TO THE RESPONDENT. THE PATIENT IS THE RESPONDENT’S…]  🞎 WIFE  🞎 HUSBAND  🞎 GIRLFRIEND  🞎 BOYFRIEND  🞎 PARTNER  🞎 SIGNIFICANT OTHER  🞎 MOTHER  🞎 MOTHER-IN-LAW  🞎 FATHER  🞎 FATHER-IN-LAW  🞎 SISTER  🞎 SISTER-IN-LAW  🞎 BROTHER  🞎 BROTHER-IN-LAW  🞎 DAUGHTER  🞎 DAUGHTER-IN-LAW  🞎 SON  🞎 SON-IN-LAW  🞎 GRANDMOTHER  🞎 GRANDFATHER  🞎 OTHER RELATIVE [SPECIFY]  🞎 FRIEND  🞎 SOMEONE ELSE [SPECIFY]  🞎 PAID HELPER (NOT A FAMILY MEMBER OR FRIEND) [INELIGIBLE, END SURVEY]  2a. So to confirm, [PATIENT’S FIRST AND LAST NAME] is your [RELATIONSHIP]?  🞎 Yes  🞎 No |
| --- |

| [TIME 2 A]  [TIME 2 B]  [TIME 2 C]   1. Is your [RELATIONSHIP] male or female? [QUESTION SPECIFICATIONS IN BOX 1]   🞎 Male  🞎 Female  **BOX 1: Q3 SPECIFICATIONS**  If Q2 = Partner, Significant Other, Friend, Someone Else, READ ALOUD Q3  If Q2 = Wife, Mother, Grandmother, Sister, Daughter, AUTOCODE Q3 as FEMALE, GO TO Q4  If Q2 = Husband, Father, Grandfather, Brother, Son, AUTOCODE Q3 as MALE, GO TO Q4 |
| --- |
| [TIME 2 A]  [TIME 2 B]  [TIME 2 C]   1. How long have you been taking part in or overseeing your [RELATIONSHIP]’s care?     🞎 Less than 3 months  🞎 At least 3 months but less than 12 months  🞎 12 months or more  [TIME 2B AND 2C: GO TO “SINCE YOUR [RELATIONSHIP] HAS BEEN HOME” SECTION INTRO AND THEN Q22] |

**IN THE HOSPITAL** *[TIME 2 A ONLY]*

**The next questions ask about your [RELATIONSHIP]’s hospital stay. When answering these questions, please only think about [his/her] stay at [HOSPITAL NAME] that ended around [DATE].**

**In this survey, a healthcare professional means a doctor, nurse, pharmacist, physical or occupational therapist, social worker, or case manager.**

| [TIME 2 A]   1. When your [RELATIONSHIP] was in the hospital, did you talk with or communicate with any healthcare professionals about [his/her] medical care?   🞎 Yes  🞎 No [GO TO “SINCE YOUR [RELATIONSHIP] HAS BEEN HOME” SECTION, Q22] |
| --- |
| [TIME 2 A]   1. When your [RELATIONSHIP] was in the hospital, did any healthcare professionals tell you or show you what you needed to do to take care of [him/her] at home?   🞎 Yes, definitely  🞎 Yes, somewhat  🞎 No  🞎 You already knew what to do |
| [TIME 2 A]   1. When your [RELATIONSHIP] was in the hospital, did any healthcare professionals make sure you understood what you needed to do to take care of [him/her] at home?   🞎 Yes, definitely  🞎 Yes, somewhat  🞎 No |
| [TIME 2 A]   1. When your [RELATIONSHIP] was in the hospital, did you practice the things you would need to do for [him/her] at home?   🞎 Yes, definitely  🞎 Yes, somewhat  🞎 No  🞎 You did not need to practice anything |
| [TIME 2 A]   1. When your [RELATIONSHIP] was in the hospital, did healthcare professionals explain things in a way you could understand?   🞎 Yes, definitely  🞎 Yes, somewhat  🞎 No |

| [TIME 2 A]   1. When your [RELATIONSHIP] was in the hospital, did healthcare professionals seem to care about you as a person?   🞎 Yes, definitely  🞎 Yes, somewhat  🞎 No |
| --- |
| [TIME 2 A]   1. When your [RELATIONSHIP] was in the hospital, did you trust healthcare professionals’ judgments about [his/her] medical care?   🞎 Yes, definitely  🞎 Yes, somewhat  🞎 No |

| [TIME 2 A]   1. Before your [RELATIONSHIP] left the hospital, did you get information about what symptoms to look out for at home?   🞎 Yes, definitely  🞎 Yes, somewhat  🞎 No |
| --- |
| [TIME 2 A]   1. Before your [RELATIONSHIP] left the hospital, did any healthcare professionals talk with you about prescription or over-the-counter medicine [he/she] should take at home?   🞎 Yes, definitely  🞎 Yes, somewhat  🞎 No [GO TO Q15]  🞎 [He/she] did not have to take any medicine at home [GO TO Q15] |
| [TIME 2 A]   1. Before your [RELATIONSHIP] left the hospital, were the possible side effects of each medicine clear to you?   🞎 Yes, definitely  🞎 Yes, somewhat  🞎 No |
| [TIME 2 A]   1. Before your [RELATIONSHIP] left the hospital, did [he/she] get written information about how to take care of [himself/herself] at home?   🞎 Yes  🞎 No [GO TO Q17] |
| [TIME 2 A]  15a. [SPANISH SURVEY VERSION ONLY] Was the written information in Spanish?  🞎 Yes  🞎 No |
| [TIME 2 A]   1. How helpful was the written information your [RELATIONSHIP] received in helping you take care of [him/her] at home?   🞎 Very helpful  🞎 Moderately helpful  🞎 A little helpful  🞎 Not at all helpful  🞎 You did not read the information |
| [TIME 2 A]   1. Before your [RELATIONSHIP] left the hospital, did [he/she] have a doctor’s appointment scheduled?   🞎 Yes  🞎 No |
| [TIME 2 A]   1. On the day your [RELATIONSHIP] left the hospital, did you think it was too soon for [him/her] to leave?   🞎 Yes, definitely  🞎 Yes, somewhat  🞎 No [GO TO “SINCE YOUR [RELATIONSHIP] HAS BEEN HOME” SECTION; Q22] |
| [TIME 2 A]   1. Was one of the reasons it was too soon because [he/she] needed more care than could be provided at home?   🞎 Yes  🞎 No |

**SINCE YOUR [RELATIONSHIP] HAS BEEN HOME**

[TIME 2 A and TIME 2 B]

**The next questions ask about the care your [RELATIONSHIP] has received since [he/she] has been home from [his/her] stay at [HOSPITAL NAME] that ended around [DATE].**

[TIME 2 C]

**The next questions ask about the care your [RELATIONSHIP] has received since we last talked with you on [DATE OF MOST RECENT T1 INTERVIEW].**

| 1. [Question only asked in Time 1 Caregiver Survey] |
| --- |
| 1. [Question only asked in Time 1 Caregiver Survey] |
| [TIME 2 A]  [TIME 2 B]   1. Since your [RELATIONSHIP] has been home, have you had contact information for a healthcare professional you could reach out to if there were any problems or questions?   [TIME 2 C]  Since we last talked with you on [DATE OF MOST RECENT T1 INTERVIEW], have you had contact information for a healthcare professional you could reach out to if there were any problems or questions?  🞎 Yes  🞎 No [GO TO Q24] |
| [TIME 2 A]  [TIME 2 B]   1. If you tried to contact them, did you get help with your problems or questions?   [TIME 2 C]  If you tried to contact them since we last talked with you, did you get help with your problems or questions?  🞎 Yes, definitely  🞎 Yes, somewhat  🞎 No  🞎 You did not try to contact them [TIME 2 C: You did not try to contact them since we last talked with you] |

| [TIME 2 A]  [TIME 2 B]   1. Since your [RELATIONSHIP] has been home, has [he/she] had to take any prescription or over-the-counter medicines?   [TIME 2 C]  Since we last talked with you on [DATE OF MOST RECENT T1 INTERVIEW], has your [RELATIONSHIP] had to take any prescription or over-the-counter medicines?  🞎 Yes  🞎 No [GO TO Q26] |
| --- |

| [TIME 2 A]  [TIME 2 B]   1. Since your [RELATIONSHIP] has been home, has there been a time when [he/she] did not take [his/her] medicine as directed?   [TIME 2 C]  Since we last talked with you, has there been a time when your [RELATIONSHIP] did not take [his/her] medicine as directed?  🞎 Yes  🞎 No |
| --- |
| [TIME 2 A]  [TIME 2 B]   1. Since your [RELATIONSHIP] has been home, has [he/she] needed to use any medical supplies or equipment - like bandages, a blood pressure monitor, oxygen, or a walker?   [TIME 2 C]  Since we last talked with you, has your [RELATIONSHIP] needed to use any medical supplies or equipment – like bandages, a blood pressure monitor, oxygen, or a walker?  🞎 Yes  🞎 No [GO TO Q28] |

| [TIME 2 A]  [TIME 2 B]   1. Since your [RELATIONSHIP] has been home, how well has [he/she] been able to use [his/her] medical supplies or equipment?   [TIME 2 C]  Since we last talked with you, how well has your [RELATIONSHIP] been able to use [his/her] medical supplies or equipment?  🞎 Very well  🞎 Moderately well  🞎 Slightly well  🞎 Not at all well  🞎 [He/She] has not used [his/her] medical supplies or equipment [Time 2 C: [He/She] has not used [his/her] medical supplies or equipment since we last talked with you] |
| --- |
| [TIME 2 A]  [TIME 2 B]   1. Since your [RELATIONSHIP] has been home, have you had to take care of a wound or surgical site for [him/her], including a catheter or ostomy?   [TIME 2 C]  Since we last talked with you, have you had to take care of a wound or surgical site for your [RELATIONSHIP], including a catheter or ostomy?  🞎 Yes  🞎 No |
| [TIME 2 A]  [TIME 2 B]   1. Since your [RELATIONSHIP] has been home, has [he/she] received help for problems with [his/her] mental or emotional health?   [TIME 2 C]  Since we last talked with you, has your [RELATIONSHIP] received help for problems with [his/her] mental or emotional health?  🞎 Yes [GO TO Q31]  🞎 No |
| [TIME 2 A]  [TIME 2 B]  [TIME 2 C]   1. Did you want [him/her] to receive help for problems with [his/her] mental or emotional health?   🞎 Yes  🞎 No |

**A healthcare professional means a doctor, nurse, pharmacist, physical or occupational therapist, social worker, or case manager.**

| [TIME 2 A]  [TIME 2 B]   1. Since your [RELATIONSHIP] has been home, have any healthcare professionals come to visit [him/her]?   [TIME 2 C]  Since we last talked with you, have any healthcare professionals come to visit your [RELATIONSHIP] at home?  🞎 Yes [GO TO Q33]  🞎 No |
| --- |
| [TIME 2 A]  [TIME 2 B]  [TIME 2 C]   1. Did you want [him/her] to have home visits from a healthcare professional?   🞎 Yes [GO TO Q34]  🞎 No [GO TO Q34] |
| [TIME 2 A]  [TIME 2 B]  [TIME 2 C]   1. How often were these home visits scheduled at times when you could be present?   🞎 Never  🞎 Sometimes  🞎 Usually  🞎 Always |
| [TIME 2 A]  [TIME 2 B]   1. Since your [RELATIONSHIP] has been home, have you talked with or communicated with any healthcare professionals about [his/her] medical care?   [TIME 2 C]  Since we last talked with you, have you talked with or communicated with any healthcare professionals about your [RELATIONSHIP]’s medical care?  🞎 Yes  🞎 No [GO TO Q40] |
| [TIME 2 A]  [TIME 2 B]   1. Since your [RELATIONSHIP] has been home, have healthcare professionals helped you manage any changes or unexpected problems with [his/her] care?   [TIME 2 C]  Since we last talked with you, have healthcare professionals helped you manage any changes or unexpected problems with your [RELATIONSHIP] ’s care?  🞎 Yes, definitely  🞎 Yes, somewhat  🞎 No  🞎 You’ve had no changes or unexpected problems with [his/her] care [Time 2 C: You’ve had no changes or unexpected problems with [his/her] care since we last talked with you] |
| [TIME 2 A]  [TIME 2 B]   1. Since your [RELATIONSHIP] has been home, have healthcare professionals explained things in a way you could understand?   [TIME 2 C]  Since we last talked with you, have healthcare professionals explained things in a way you could understand?  🞎 Yes, definitely  🞎 Yes, somewhat  🞎 No |
| [TIME 2 A]  [TIME 2 B]   1. Since your [RELATIONSHIP] has been home, have healthcare professionals seemed to care about you as a person?   [TIME 2 C]  Since we last talked with you, have healthcare professionals seemed to care about you as a person?  🞎 Yes, definitely  🞎 Yes, somewhat  🞎 No |
| [TIME 2 A]  [TIME 2 B]   1. Since your [RELATIONSHIP] has been home, have you trusted healthcare professionals’ judgments about your [RELATIONSHIP]’s medical care?   [TIME 2 C]  Since we last talked with you, have you trusted healthcare professionals’ judgments about your [RELATIONSHIP]’s medical care?  🞎 Yes, definitely  🞎 Yes, somewhat  🞎 No |
| [TIME 2 A]  [TIME 2 B]   1. Since your [RELATIONSHIP] has been home, has a healthcare professional told you something that went against what another healthcare professional told you?   [TIME 2 C]  Since we last talked with you, has a healthcare professional told you something that went against what another healthcare professional told you?  🞎 Yes, definitely  🞎 Yes, somewhat  🞎 No |

**BOX 2: OVERALL QUALITY SECTION SPECIFICATIONS**

TIME 2 A:

IF Q5 = NO/DK/RF 🡪 READ INTRO, THEN SKIP TO Q41

IF Q5 = YES 🡪 READ INTRO, COMPLETE ALL QUESTIONS IN THIS SECTION (Q40-Q43)

TIME 2 B & TIME 2 C:

READ INTRO, THEN SKIP TO Q41

**OVERALL QUALITY OF CARE RECEIVED**

**Now I am going to ask you some overall questions about your experiences.**

| [TIME 2 A]   1. Overall, how would you rate the hospital in preparing you to take care of your [RELATIONSHIP] at home?     🞎 Excellent  🞎 Very good  🞎 Good  🞎 Fair  🞎 Poor |
| --- |
| [TIME 2 A]  [TIME 2 B]   1. Overall, how would you rate your ability to take care of your [RELATIONSHIP] since [he/she] has been home?   [TIME 2 C]  Overall, how would you rate your ability to take care of your [RELATIONSHIP] since the we last talked with you?  🞎 Excellent  🞎 Very good  🞎 Good  🞎 Fair  🞎 Poor |
| [TIME 2 A]  [TIME 2 B]   1. Overall, how would you rate the care your [RELATIONSHIP] has received from healthcare professionals since [he/she] has been home?   [TIME 2 C]  Overall, how would you rate the care your [RELATIONSHIP] has received from healthcare professionals since we last talked with you?  🞎 Excellent  🞎 Very good  🞎 Good  🞎 Fair  🞎 Poor  🞎 [He/she] has not received care from any healthcare professionals [TIME 2 C: [He/she] has not received care from any healthcare professionals since we last talked with you] |
| [TIME 2 A]  [TIME 2 B]  [TIME 2 C]   1. Overall, have healthcare professionals been there for you as much as you needed?   🞎 Yes, definitely  🞎 Yes, somewhat  🞎 No |

**YOUR AMOUNT OF STRESS**

**The next few questions ask what it has been like for you to take care of your [RELATIONSHIP].**

| [TIME 2 A]  [TIME 2 B]   1. Since your [RELATIONSHIP] has been home, how much effort has it taken for you to care for [him/her]?   [TIME 2 C]  Since we last talked with you, how much effort has it taken for you to care for your [RELATIONSHIP] at home?  🞎 No effort  🞎 A little effort  🞎 A moderate amount of effort  🞎 A lot of effort |
| --- |
| [TIME 2 A]  [TIME 2 B]   1. Since your [RELATIONSHIP] has been home, how stressful has it been to care for [him/her]?   [TIME 2 C]  Since we last talked with you, how stressful has it been to care for your [RELATIONSHIP] at home?  🞎 Not at all stressful  🞎 Somewhat stressful  🞎 Moderately stressful  🞎 Very stressful |
| [TIME 2 A]  [TIME 2 B]   1. From the time your [RELATIONSHIP] left [HOSPITAL NAME] around [DATE] until now, how has taking care of [him/her] changed? Is it…   [TIME 2 C]  From the time we last talked with you until now, how has taking care of your [RELATIONSHIP] changed? Is it…  A lot easier?  A little easier?  About the same?  A little harder?  A lot harder? |

**ABOUT YOU**

**I have just a few more questions for you before we finish.**

| [TIME 2 A]  [TIME 2 B]  [TIME 2 C]   1. Do you and your [RELATIONSHIP] live together?   🞎 Yes  🞎 No |
| --- |
| [TIME 2 A]  [TIME 2 B]  [TIME 2 C]   1. Do other people help care for your [RELATIONSHIP]?   🞎 Yes  🞎 No |
| [TIME 2 A]  [TIME 2 B]  [TIME 2 C]   1. Are you male or female?   🞎 Male  🞎 Female |
| [TIME 2 A]  [TIME 2 B]  [TIME 2 C]   1. What is the highest grade or level of school you have completed?     🞎 8th grade or less  🞎 Some high school, but did not graduate  🞎 High school graduate or GED  🞎 Some college or 2-year degree  🞎 4-year college graduate  🞎 More than a 4-year college degree |
| [TIME 2 A]  [TIME 2 B]  [TIME 2 C]   1. Currently, what is your employment status?   🞎 full-time for pay  🞎 full-time unpaid  🞎 part-time for pay  🞎 part-time unpaid  🞎 Not working or Retired |

**Those are all the questions that I have. Thank you very much for your help with this survey.**

**Have a nice (day/evening). Goodbye.**
